# Next-generation muscle-directed gene therapy by in silico vector design

**DOI:** 10.1038/s41467-018-08283-7

**Published:** 2019-01-30

**Authors:** S. Sarcar, W. Tulalamba, M. Y. Rincon, J. Tipanee, H. Q. Pham, H. Evens, D. Boon, E. Samara-Kuko, M. Keyaerts, M. Loperfido, E. Berardi, S. Jarmin, P. In’t Veld, G. Dickson, T. Lahoutte, M. Sampaolesi, P. De Bleser, T. VandenDriessche, M. K. Chuah

**Affiliations:** 1 Department of Gene Therapy & Regenerative Medicine, Vrije Universiteit Brussel (VUB), Brussels, 1090 Belgium; 2 Centro de Investigaciones, Fundacion Cardiovascular de Colombia, Floridablanca, 681004 Colombia; 3 Center for Molecular & Vascular Biology, Department of Cardiovascular Sciences, University of Leuven, 3000 Leuven, Belgium; 4 Nuclear Medicine Department, UZ Brussel & In vivo Cellular and Molecular Imaging Lab, Vrije Universiteit Brussel (VUB), Brussels, 1090 Belgium; 5 Translational Cardiomyology Laboratory, Embryo and Stem Cell Biology Unit, Department of Development and Regeneration, University of Leuven, Leuven, 3000 Belgium; 6 School of Biological Sciences, Royal Holloway, University of London, Egham, Surrey, TW20 0EX UK; 7 Department of Pathology, Diabetes Research Center, Vrije Universiteit Brussel (VUB), Brussels, 1090 Belgium; 8 VIB Center for Inflammation Research and Department of Biomedical Molecular Biology, Ghent University, Ghent, 9052 Belgium

## Abstract

There is an urgent need to develop the next-generation vectors for gene therapy of muscle disorders, given the relatively modest advances in clinical trials. These vectors should express substantially higher levels of the therapeutic transgene, enabling the use of lower and safer vector doses. In the current study, we identify potent muscle-specific transcriptional *cis*-regulatory modules (*CRMs*), containing clusters of transcription factor binding sites, using a genome-wide data-mining strategy. These novel muscle-specific *CRMs* result in a substantial increase in muscle-specific gene transcription (up to 400-fold) when delivered using adeno-associated viral vectors in mice. Significantly higher and sustained human micro-dystrophin and follistatin expression levels are attained than when conventional promoters are used. This results in robust phenotypic correction in dystrophic mice, without triggering apoptosis or evoking an immune response. This multidisciplinary approach has potentially broad implications for augmenting the efficacy and safety of muscle-directed gene therapy.

## Abstract (web summary)

Adeno-associated viral vectors (AAV) are being developed for gene therapy of skeletal muscle, but it is a challenge to achieve robust gene expression. Here, the authors identify muscle-specific cisregulatory elements that lead to a substantial increase in micro-dystrophin and follistatin expression, resulting in a safe and sustainable rescue of the dystrophic phenotype in mouse models.

## Introduction

Hereditary muscle disorders are characterized by significant morbidity and mortality due to skeletal muscle and cardiac dysfunction^1^. Most of these diseases lack effective treatment, which underscores their unmet medical need. In view of recent clinical successes^2–5^, gene therapy offers promising therapeutic perspectives for many genetic diseases, including muscle disorders. Most importantly, muscle-directed gene therapy constitutes the basis of the first regulatory approved gene therapy product^6–9^.

Most common hereditary muscle disorders are caused by single gene defects. In particular, Duchenne muscular dystrophy (DMD) affects 1 in 3500 live newborn males and is caused by mutations in the dystrophin (*DYS*) gene^10^. Patients typically die at an early age from cardiopulmonary failure after progressive muscle deterioration^11^. Long-term expression and therapeutic effects have been reported after gene therapy using adeno-associated viral vectors (AAVs) in animal models^12–15^. Typically, for gene therapy of DMD, truncated versions of the dystrophin gene are used (i.e., micro-dystrophin)^16,17^ that can readily be accommodated into these AAV vectors. In addition to correcting muscle diseases per se, the muscle is also an attractive target for delivery of secreted proteins into the circulation after gene therapy given its secretory capacity^18,19^.

Despite its promise, it has been particularly challenging to achieve robust and widespread expression of a given therapeutic gene in the skeletal muscle^18,20–22^. For instance, clinical trials for DMD establish proof of concept that targeting the muscle by gene therapy is feasible, though the overall therapeutic benefits were limited^20,23^. Moreover, muscle-directed oligonucleotide-mediated exon-skipping strategies did not yet yield the expected outcome in pivotal trials in patients suffering from DMD^24,25^, despite increased dystrophin-expression^26,27^. Though increasing the vector doses may result in more efficient gene transfer, this concomitantly increases the risk of triggering inadvertent immune reactions^28,29^. Hence, to overcome these limitations, it is mandatory to develop more potent gene therapy vectors containing novel promoters that outperform conventional ones^22,30,31^ and that consequently allow for high and widespread muscle-specific expression at lower and safer vector doses.

In the current study, we use genome-wide data-mining to identify robust transcriptional *cis*-regulatory modules (*CRMs*) that are effective in boosting transcriptional targeting up to 400-fold in skeletal muscle. This multidisciplinary approach provides new insights into regulatory motifs and their relative strength in conferring muscle-specific transcriptional control. These novel muscle *CRMs* increase expression of micro-dystrophin (MD1)^16,17^ and follistatin (FST344), a known myostatin inhibitor^32^, after gene therapy with serotype 9 adeno-associated viral vectors (AAV9)^33,34^. This results in sustained phenotypic correction in dystrophic mice without any discernable immune complications.

## Results

### Computational identification of *Sk-CRM*

**Fig. 1:**
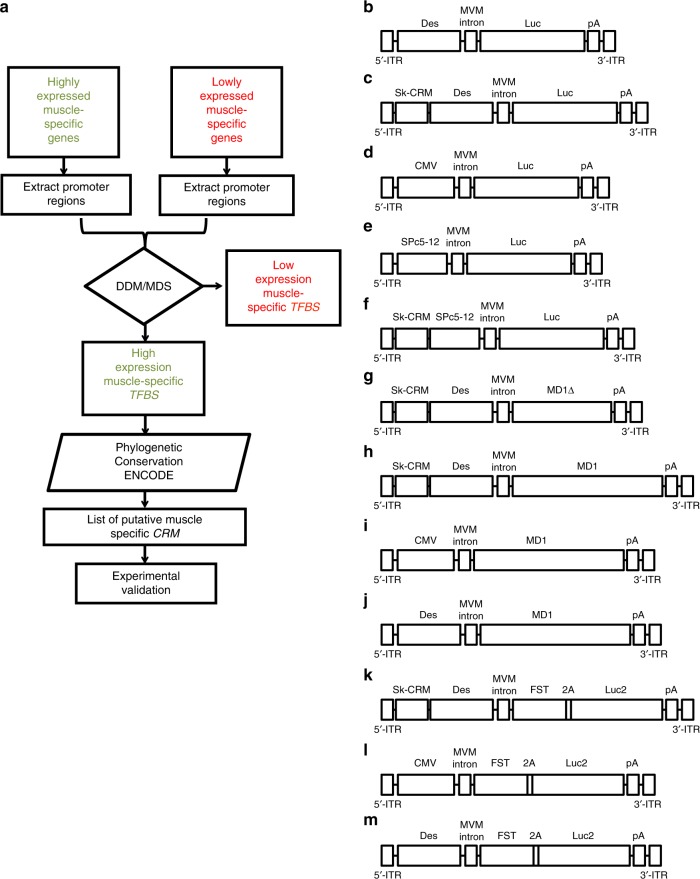
Flow diagram for the identification of muscle-specific *cis*-regulatory modules (*CRMs*) and AAV constructs for in vivo validation. **a** A computational approach was used to identify the *Sk-CRMs* involving the following five steps: (1) identification of tissue-specific genes that are highly and lowly expressed based on statistical analysis of micro-array expression data of normal human tissues; (2) extraction of the corresponding promoter sequences from publicly available databases; (3) identification of the *CRMs* and the transcription factor binding sites (*TFBS*) they contain using a differential distance matrix (DDM)-multidimensional scaling (MDS) approach; (4) search for evolutionary conserved clusters of *TFBS* (i.e., *CRM*) of the highly expressed genes (5) ENCODE filtering. The identified *Sk-CRMs* were subsequently included in an expression construct and validated in vivo by testing whether they increased promoter activity. **b**–**m** Schematic representation of all the different AAV vectors encoding either the reporter or therapeutic genes. The different expression cassettes were packaged in an adeno-associated virus vector flanked by inverted terminal repeats (ITR) from AAV serotype 2 (AAV2) and produced with an AAV serotype 9 (AAV9) capsid. The expression cassette further comprises the Minute Virus of Mouse (MVM) intron and a Simian virus 40 (SV40) polyadenylation signal (*pA*). **b** The scAAV-Des-Luc vector is a self-complementary AAV vector (scAAV) containing the luciferase (*Luc)* gene driven from the desmin (*Des*) promoter without the CRM used as a control **c** the scAAV-Sk-CRM-Des-Luc vectors contain the *Sk-CRM 1-7* cloned upstream the *Des* promoter. **d** The scAAV-CMV-Luc vector uses the *CMV* promoter to drive the *Luc* gene. **e** The scAAV-SPc5-12-Luc vector has the same vector configuration as in **b** where the *Des* promoter was replaced by the *SPc5-12* promoter. **f** The scAAV-Sk-CRM4-SPc5-12-Luc vector contains the *Sk-CRM4* element cloned upstream of the *SPc5-12* promoter driving the expression of the *Luc* gene. The single-stranded AAV (ssAAV) vectors were used to deliver therapeutic genes **g**–**j** micro-dystrophin (*MD1*Δ or *MD1*) and **k**–**m** the follistatin (*FST344*), respectively, with *SkCRM4/Des* chimeric promoter or CMV and *Des* conventional promoters. The cognate FST protein is encoded by a *FST-2A-Luc* polycistronic transcript

To design robust muscle-specific gene therapy vectors, we relied on a multistep computational approach (Fig. 1a) that allowed us to identify novel evolutionary conserved skeletal muscle-specific *cis*-regulatory modules (designated as *Sk-CRMs*) associated with genes that are highly expressed in the skeletal muscle (Table 1 and Supplementary Table 1). Using this computational approach, we have identified seven different *Sk-CRMs* based on human sequences ranging from sizes 344 bp to 519 bp (Table 1, Supplementary Table 1, and Supplementary Fig. 1). These *Sk-CRMs* comprised binding sites for seven different transcription factors (TFs) including E2A, CEBP, LRF, MyoD, SREBP, Tal1, PPAR (Table 1; Supplementary Fig. 1, and Supplementary Table 2). The *Sk-CRM* elements (i.e., *Sk-CRM1* to *Sk-CRM7*) corresponded to transcription factor binding site (*TFBS*) clusters in the promoters or introns of the following muscle-specific genes: *ATP2A1 (Sk-CRM1), TNNI1 (Sk-CRM2, Sk-CRM3), MYLPF (Sk-CRM4); MYH1 (Sk-CRM5), TPM3 (Sk-CRM6), ANKRD2 (Sk-CRM7)* (Table 1, Supplementary Table 1, and Supplementary Fig. 1). Several *Sk-CRMs* contain identical *TFBS* but each *Sk-CRM* is unique with respect to the specific *TFBS* arrangement. These distinct *Sk-CRMs*, therefore, contained a molecular signature that is characteristic of genes that are highly expressed in the muscle. The selected *Sk-CRMs* were relatively conserved in evolution (Supplementary Fig. 1), suggesting strong selective pressure to maintain these particular *TFBS* combinations to enable high muscle-specific expression. The use of these evolutionary conserved human *Sk-CRMs* increased the likelihood that their potency and specificity is preserved following clinical translation.

**Table 1:** Sequence and details of the *Sk-CRMs*

Name	Genes	Length (bp)	TFBS
SK-CRM1	*ATP2A1*	495	E2A, CEBP, LRF, MyoD
SK-CRM2	*TNNI1*	344	E2A, CEBP, LRF, MyoD, SREBP
SK-CRM3	*TNNI1*	430	E2A, CEBP, LRF, MyoD, SREBP, Tal1.
SK-CRM4	*MYLPF*	435	E2A, CEBP,LRF, MyoD, SREBP
SK-CRM4^a^		171	CEBP, E2A, LRF
SK-CRM4^b^		51	CEBP, E2A, LRF
SK-CRM4^c^		60	E2A, LRF, MyoD
SK-CRM4^d^		41	LRF, MyoD
SK-CRM4^e^		120	CEBP, E2A, LRF
SK-CRM5	*MYH1*	474	PPAR, CEBP, LRF, SREBP
SK-CRM6	*TPM3*	519	E2A, CEBP, LRF, MyoD, SREBP
SK-CRM7	*ANKRD2*	372	E2A, CEBP, MyoD

### In vivo identification of robust muscle-specific *Sk-CRM*

**Fig. 2:**
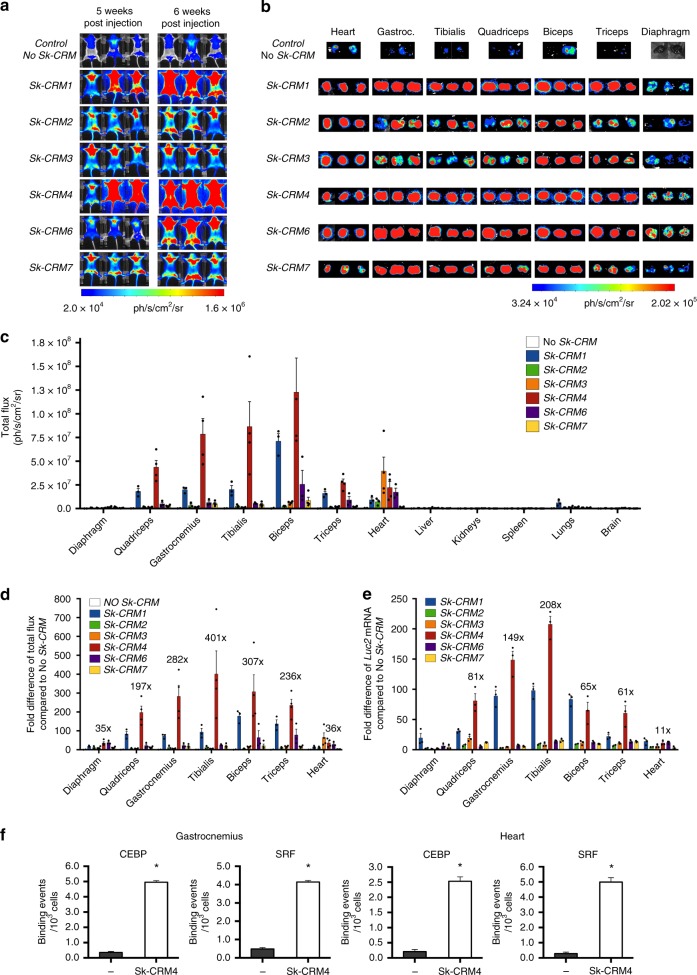
Screening and validation of novel hyperactive *Sk-CRMs*. CB17/IcrTac/Prkdc^scid^ mice were intravenously injected with 5 × 10^9^ vg per neonatal mouse. **a** In vivo whole-body bioluminescence imaging of mice injected intravenously with scAAV9-Des-Luc2 (control, no *Sk-CRM*), scAAV9-Sk-CRM1-Des-Luc (*Sk-CRM1*), scAAV9-Sk-CRM2-Des-Luc (*Sk-CRM2*), scAAV9-Sk-CRM3-Des-Luc (*Sk-CRM3*), scAAV9-Sk-CRM4-Des-Luc (*Sk-CRM4*), scAAV9-Sk-CRM6-Des-Luc (*Sk-CRM6*), or scAAV9-Sk-CRM7-Des-Luc (*Sk-CRM7*) vector were shown based on a color scale from 2.0e + 04 (blue) ph/s/cm^2^/sr to 1.59e + 06 (red) ph/s/cm^2^/sr at week 5 and 6 post vector injection. Photon emission was measured dynamically during 7 min in a supine position. **b** Ex vivo bioluminescence imaging of individual organs harvested at week 7 post vector injection was represented on a color scale with luciferase intensities ranging from 3.23e + 04 (blue) ph/s/cm^2^/sr to 2.05e + 05 (red) ph/s/cm^2^/sr. The bioluminescence signal was quantified for 5 min. **c** A hand-drawn region of interest (ROI) was used for every individual tissue. Luciferase expression from the individual muscles was measured as total flux, expressed in photons/sec/cm^2^/sr. **d** Fold-difference of the total flux measured from the different muscles upon ex vivo bioluminescence imaging with respect to the control (construct without *Sk-CRM*). **e** Fold-difference in *Luc* mRNA expression levels from the different muscles, measured by qRT-PCR from total RNA extracted from biopsies of the indicated tissues. Results were presented as mean ± standard error of the mean, fold-difference in *Luc* mRNA expression with respect to the control (construct without *Sk-CRM*). **f** Chromatin immune-precipitation (ChIP) assay for the heart and gastrocnemius muscle of mice injected with scAAV9-Sk-CRM4-Des-Luc (5 × 10^9^ vg/mouse). Antibodies specific for CEBP and SRF and PCR primers specific for the corresponding *TFBS* were used. PCR primers were designed to amplify a region corresponding to *Sk-CRM4* that binds CEBP and SRF and an untranscribed region on chromosome 17 was used as a negative control. Binding events for 10^3^ cells were determined for each of the corresponding primer pairs. The black bars represent the negative control and the white bars correspond to *Sk-CRM4*; heart (left panel), gastrocnemius (right panel). Significant differences compared to the negative control were indicated (*t*-test, *p ≤ 0.05, mean + s.e.m.; *n* = 2–4)

An in vivo screening of these *Sk-CRMs* was subsequently performed to identify the most robust elements. To achieve this, we cloned the different *Sk-CRMs* upstream of a desmin (*Des*) promoter (Fig. 1b) that drove the expression of a luciferase (*Luc*) reporter gene. The *Des* promoter was chosen since it is known to confer relatively high levels of skeletal muscle and heart-specific transgene expression^35^. The constructs were packaged using AAV serotype 9 (AAV9) to maximize skeletal muscle and cardiac-specific gene transfer^36–38^. The scAAV9-Sk-CRM-Des-Luc vectors containing either *Sk-CRM1, Sk-CRM2, Sk-CRM3, Sk-CRM4, Sk-CRM6*, or *Sk-CRM7* (Fig. 1c) were then intravenously injected at a dose of 5 × 10^9 ^vg/mouse into neonatal CB17/IcrTac/Prkdc^scid^ mice. Bioluminescence imaging revealed that 6 out of 6 (100%) (Fig. 2a–c) different *Sk-CRMs* that were tested in vivo significantly augmented expression of the luciferase reporter gene from the *Des* promoter in distinct skeletal muscle groups but not in other organs. Most importantly, the *Sk-CRM4* element resulted in an unprecedented and significant 200 to 400-fold increase (*t*-test; *p* < 0.05) in luciferase expression (based on total photon flux) from the *Des* promoter in different muscle groups (i.e., gastrocnemius, tibialis, quadriceps, biceps, and triceps) (Fig. 2d), compared to controls without *Sk-CRM4*. This was consistent with a significant (*t*-test; *p* < 0.01) 60 to 200-fold increase in *Luc* mRNA expression in these different muscle groups when the *Sk-CRM4* was used in combination with the *Des* promoter (Fig. 2e). Linear regression analysis confirmed a strong correlation between the luciferase activity and the *Luc* mRNA levels in the different muscle groups (correlation coefficient *R*^2^ = 0.8). Interestingly, the *Sk-CRM4* element also increased luciferase expression in the diaphragm (i.e., 35-fold) (Fig. 2b–d) but to a lesser extent than in the other skeletal muscles. This difference likely reflects the lower intrinsic activity of the *Des* promoter in the diaphragm (Fig. 2b). Alternatively, the reduced transduction efficiency with AAV9 in diaphragm compared to most of the other muscle groups may also have contributed to the difference in luciferase expression (Supplementary Fig. 2). Moreover, the selected *Sk-CRMs* also significantly increased expression in the heart, reflecting the tropism of AAV9 and the specificity of the *Des* promoter (Fig. 2b, c). In particular, the most robust and significant (*t*-test; *p* < 0.05) increase in cardiac expression was achieved with the *Sk-CRM4* (i.e., 36-fold) or *Sk-CRM3* elements (i.e., 65-fold) (Fig. 2d).

Despite the relatively broad tissue-tropism of AAV9 (Supplementary Fig. 2), luciferase reporter gene expression was mainly restricted to skeletal muscle and heart. In particular, liver, kidney, spleen, and the brain showed no or only limited luciferase expression with any of the *Sk-CRMs* (Fig. 2c and Supplementary Fig. 3). Nevertheless, low-level expression was apparent in the lung (Supplementary Fig. 3), albeit substantially less compared to that in skeletal muscle or heart (Fig. 2c), particularly when the most robust *Sk-CRM4* was employed.

The computational algorithm predicted that *Sk-CRM4* contained clusters of *TFBS*, including CEBP and SRF (Table 1, Supplementary Tables 1 and 2, and Supplementary Fig. 1). We, therefore, experimentally confirmed the binding of CEBP and SRF, on the most potent *Sk-CRM4* element by chromatin immunoprecipitation (ChIP) using gastrocnemius muscle and heart from mice that were injected with the scAAV9-Sk-CRM4-Des-Luc vector. In particular, the ChIP assay revealed a significant 23-fold enrichment in the case of both CEBP and SRF on the *Sk-CRM4* element over the negative control for the gastrocnemius muscle. Similarly, a specific 12-fold enrichment in case of CEBP and a 26-fold enrichment for SRF on the *Sk-CRM4* element over the negative control were apparent in the heart (Fig. 2f). Taking into account that the computational motif search tool estimates the overall probability of a TF binding to a given promoter region, these ChIP assay results suggest binding of the respective TFs on a promoter region of the *MYLPF* gene, that encompasses the corresponding TFBS elements. Hence, these ChIP assay results suggest that TF binding on the corresponding *TFBS* elements likely contributed to the increased transcriptional activity, which in turn resulted in higher protein expression levels.

### In vivo validation of *Sk-CRM4* fragments

**Fig. 3:**
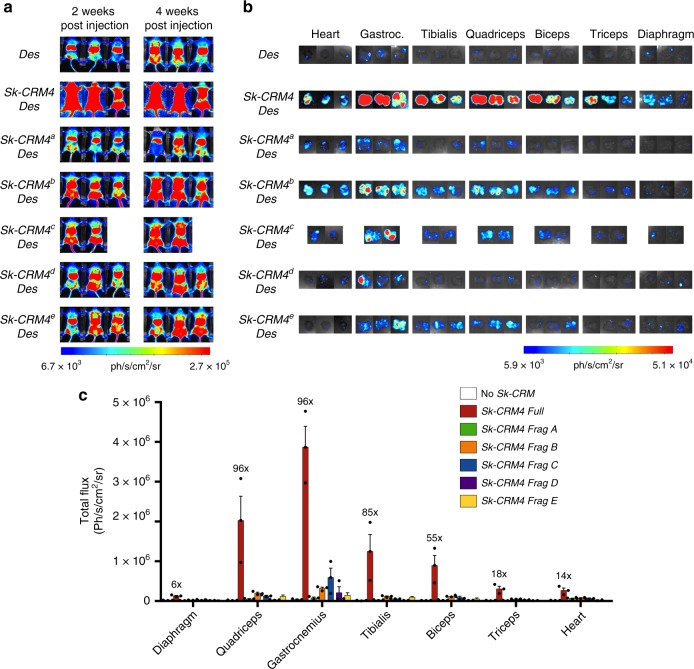
Screening and validation of *Sk-CRM4* fragments. **a** In vivo bioluminescence imaging at week 2 and week 4 post vector injection and quantification of luciferase expression from the different individual organs of adult CB17/IcrTac/Prkdc^scid^ mice injected intravenously with scAAV9-Des-Luc2 (control, no *Sk-CRM*), scAAV9-Sk-CRM4^a^-Des-Luc, scAAV9-Sk-CRM4^b^-Des-Luc, scAAV9-Sk-CRM^c^-Des-Luc, scAAV9-Sk-CRM4^d^-Des-Luc, and scAAV9-Sk-CRM4^e^-Des-Luc vectors at a dose of 1 × 10^10^ vg per mouse. The corresponding color scale from 6.70e + 03 (blue) ph/s/cm^2^/sr to 2.70e + 05 (red) ph/s/cm^2^/sr was shown. Photon emission was measured dynamically during 7 min in a supine position. **b** Ex vivo bioluminescence imaging of individual organs such as the heart and the different muscle groups harvested at 6 weeks post vector injection was represented on a color scale with luciferase intensities ranging from 5.92e + 03 (blue) ph/s/cm^2^/sr to 5.08e + 04 (red) ph/s/cm^2^/sr. The bioluminescence signal was quantified for 5 min. **c** Luciferase expression from the individual tissues was measured as total flux, expressed in photons/sec/cm^2^/sr. The fold-difference of the total flux was indicated for each *Sk-CRM4* fragments relative to that of the control group containing the *Des* promoter without any *Sk-CRM*. The total flux data were displayed as mean + s.e.m. (*n* = 3)

Subsequently, we assessed whether smaller fragments of the computationally defined *Sk-CRM4* element, were capable of producing an effect similar to what could be achieved with the full-length *Sk-CRM4*. Moreover, since the initial screening was conducted in neonates (Fig. 2), it was important to also confirm the *Sk-CRM4* effect in adult mice (Fig. 3). These *Sk-CRM4* fragments (designated as *Sk-CRM4*^*a*^, *Sk-CRM4*^*b*^*, Sk-CRM4*^*c*^*, Sk-CRM4*^*d*^, and *Sk-CRM4*^*e*^) were identified by selecting out smaller fragments within the full-length *Sk-CRM4* containing dense clusters of *TFBS* (Table 1, Supplementary Fig. 1, and Supplementary Table 1). The scAAV9-Sk-CRM-Des-Luc vectors (containing either the full-length *Sk-CRM4* or its *Sk-CRM4*^*a*^, *Sk-CRM4*^*b*^*, Sk-CRM4*^*c*^*, Sk-CRM4*^*d*^, and and *Sk-CRM4*^*e*^ fragments) were then intravenously injected at a dose of 10^10^ vg/mouse into adult CB17/IcrTac/Prkdc^scid^ mice. The scAAV9-Des-Luc vector was used as control. Bioluminescence imaging revealed that the full-length *Sk-CRM4* resulted in a significant and robust increase in skeletal muscle-specific expression (up to 100-fold), compared to the control vector without *Sk-CRM4*, in adult mice with no increased expression in other non-target tissues (Fig. 3 and Supplementary Fig. 4). Though the five different *Sk-CRM* fragments significantly augmented expression in the different skeletal muscle groups and in the heart, the overall effect was more modest (typically 3 to 9-fold) than what could be achieved with the full-length *Sk-CRM4* element. This indicates that it is the specific combination of all *TFBS* clusters within the full-length *Sk-CRM4* element that is required to confer high levels of transgene expression in the targeted skeletal muscles and heart.

### Comparison of promoter strength with conventional promoters

**Fig. 4:**
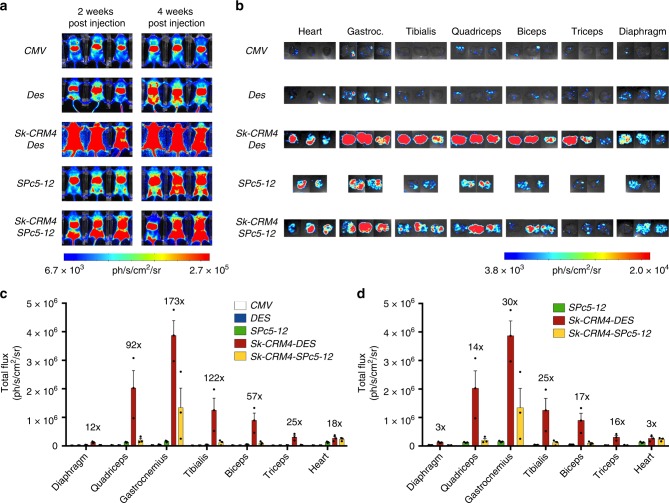
Comparison of *Sk-CRM*/promoter combinations with conventional promoters. **a** In vivo bioluminescence imaging at week 2 and week 4 post vector injection in adult CB17/IcrTac/Prkdc^scid^ mice intravenously injected with the different scAAV9 luciferase vectors containing the different promoters (*CMV, Des, Sk-CRM4/Des, SPc5-12, Sk-CRM4/SPc 5-12*) at a dose of 1 × 10^10 ^vg per mouse. A color scale from 6.7e + 03 (blue) ph/s/cm^2^/sr to 2.7e + 05 (red) ph/s/cm^2^/sr was shown. Photon emission was measured dynamically during 7 min. **b** Ex vivo bioluminescence imaging of individual tissues harvested at 6-week post vector injection was represented on a color scale with luciferase intensities ranging from 3.80e + 03 (blue) ph/s/cm^2^/sr to 2.02e + 04 (red) ph/s/cm^2^/sr. The bioluminescence signal was quantified for 5 min. **c** Luciferase expression from the individual tissues was measured as total flux, expressed in ph/s/cm^2^/sr. The fold-difference of the total flux of the *SkCRM4/Des* chimeric promoter was indicated relative to the *CMV* promoter (without any *Sk-CRM)*. The luciferase signal were showed as mean + s.e.m. (*n* = 3)

The performance of the *Sk-CRM4/Des* chimeric promoter was subsequently compared with that of *CMV* and the synthetic *SPc5-12* promoter, commonly used for muscle gene therapy. We also combined the most robust *Sk-CRM4* element with the *SPc5-12* promoter to assess whether it can further increase its strength, as in the case of the *Des* promoter. The scAAV9-Sk-CRM4-Des-Luc, scAAV9-Des-Luc, scAAV9-Sk-CRM4-SPc5-12-Luc, scAAV9-SPc5-12-Luc, or scAAV9-CMV-Luc vectors (Fig. 1b–f) were injected intravenously at a dose of 10^10 ^vg/mouse in adult CB17/IcrTac/Prkdc^scid^ mice. Bioluminescence analysis in vivo (Fig. 4a) or ex vivo (Fig. 4b) on the isolated muscles revealed that the *Sk-CRM4/Des* combination was the most robust *Sk-CRM*/promoter combination. In particular, compared to *CMV*, a significant 25 to 173-fold increase (*t*-test; *p* < 0.01) in transgene expression from the different skeletal muscles could be attained. Moreover, a significant 12 and 18-fold increase (*t*-test; *p* < 0.05) in luciferase expression could be achieved with *Sk-CRM4/Des* in the diaphragm and heart, respectively (Fig. 4c). Most importantly, *Sk-CRM4/Des* also outperformed the *SPc5-12* and the *Sk-CRM4/SPc5-12* promoters (Fig. 4c). These results suggested that the *Sk-CRM4* element did not only increase expression from the *Des* promoter but also boosted the performance of the *SPc5-12* promoter in skeletal muscle and heart, although to a lesser extent than in the case of the *Des* promoter. In particular, when comparing the *Sk-CRM4/SPc5-12* chimeric promoter with the *SPc5-12* promoter, a 15- to 30-fold increase in expression was attained (based on total flux data from different muscles not including diaphragm). This indicates that the *Sk-CRM4* element has the potential to augment expression from different muscle-specific promoters, the extent of which varies depending on the intrinsic strength of those promoters.

### Therapeutic validation in a dystrophic mouse model

**Fig. 5:**
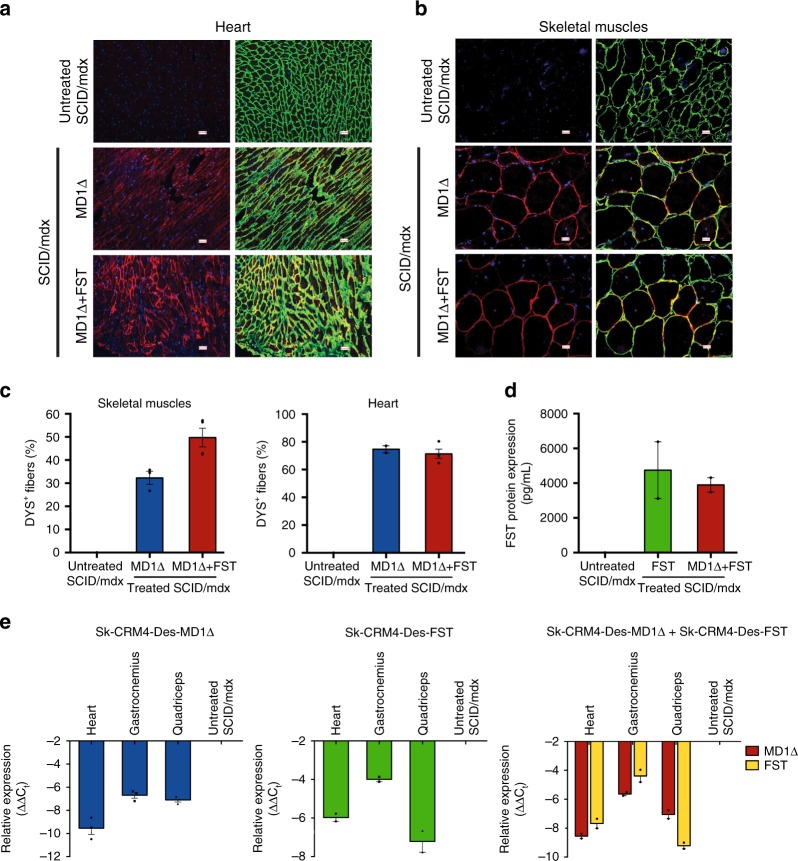
Human MD1 and human FST expression analysis in immunodeficient dystrophic SCID/mdx mice. The ssAAV9-Sk-CRM4-Des-MD1Δ and ssAAV9-Sk-CRM4-Des-FST vectors were injected either alone (indicated as MD1Δ, FST) or in combination (indicated as MD1Δ + FST) in SCID/mdx mice at a dose of 2 × 10^10^ vg/mouse. **a**, **b** Immunofluorescence staining for laminin (LAM, in green), human dystrophin (DYS, in red) and DAPI nuclear staining (in blue) was performed on sections from heart (**a**) and skeletal muscles (**b**) of treated and untreated control SCID/mdx mice. The scale bars indicate 100 μm. **c** Quantification of the DYS^+^ fibers detected out of the total number of LAM^+^ fibers in the heart and skeletal muscle tissue sections from treated and untreated control SCID/mdx mice. **d** Quantification of human FST protein (in pg/50 mg of skeletal muscle tissue) as determined by ELISA. **e** Human *MD1Δ* and *FST* mRNA quantification in the heart and the skeletal muscles (i.e., gastrocnemius, quadriceps) of treated and untreated control SCID/mdx mice, expressed relative to the murine housekeeping gene Gapdh by qRT-PCR. Mice were injected at 4 weeks and euthanized 22 weeks later. The data were represented as mean + s.e.m. (*n* = 2–4)

We subsequently validated the performance of the *Sk-CRM4/Des* chimeric promoter in a preclinical gene therapy setting. We, therefore, generated AAV9 vectors expressing either a truncated codon-usage optimized human micro-dystrophin (*MD1Δ*) (Fig. 1g) or a human follistatin cDNA (*FST344*) (Fig. 1k). Injection of ssAAV9-Sk-CRM4-Des-MD1Δ by itself or in combination with ssAAV9-Sk-CRM4-Des-FST in SCID/mdx mice resulted in widespread MD1 expression in heart and skeletal muscles (Fig. 5a, b), consistent with a significant percentage (Fig. 5c) of dystrophin-positive fibers (DYS^+^) in both heart and skeletal muscle compared to untreated control SCID/mdx mice. Similarly, injection of ssAAV9-Sk-CRM4-Des-FST by itself or in combination with AAV9-Sk-CRM4-Des-MD1Δ resulted in significantly higher FST protein levels (Fig. 5d) expression in skeletal muscle compared to untreated control SCID/mdx mice. These results were consistent with significantly elevated levels of *MD1Δ* or *FST* transcripts in transduced skeletal muscle and heart of SCID/mdx mice (Fig. 5e).

**Fig. 6:**
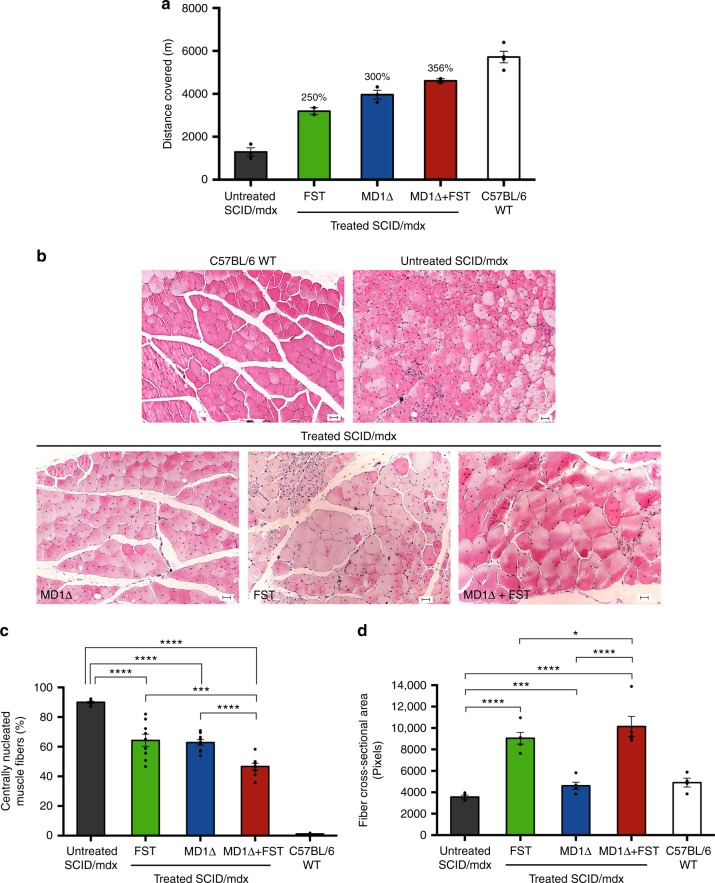
Phenotypic correction of muscular dystrophy in SCID/mdx mice. **a** SCID/mdx mice injected with ssAAV9-Sk-CRM4-Des-MD1Δ only, ssAAV9-Sk-CRM4-Des-FST only, and ssAAV9-Sk-CRM4-Des-MD1Δ with ssAAV9-Sk-CRM4-Des-FST combination therapy (2 × 10^10^ vg/mouse of each vector) were subjected to a treadmill assay. Physical performance of the 20-week-old SCID/mdx mice treated with the different therapeutic vectors compared to the untreated age-matched control SCID/mdx mice was determined by measuring the distance covered. Results were presented as mean ± standard error of the mean, (% increase in distance covered relative to untreated SCID/mdx was indicated. **b**–**d** Improvement of pathophysiological properties in SCID/mdx mice injected with ssAAV9-Sk-CRM4-Des-MD1Δ, ssAAV9-Sk-CRM4-Des-FST, ssAAV9-Sk-CRM4-Des-MD1Δ, and ssAAV9-Sk-CRM4-Des-FST combination therapy. **b** Hematoxylin and eosin staining of 5 μm thick transverse sections of the tibialis anterior muscle from mice treated with the different therapeutic vectors were compared with those of age-matched control C57BL/6 and SCID/mdx mice treated with PBS. The scale bars indicate 50 μm. **c** Graphical representation of the % of central nucleation in muscle fibers from treated sections versus the age-matched non-treated sections. Results were presented as mean ± standard error of the mean, ***p < 0.001 and ****p < 0.0001 using Student’s *t*-test (*n* = 4–9). **d** Muscle fiber cross-sectional area (expressed in pixels) of each of the different groups of SCID/mdx mice treated with the different therapeutic vectors (at a dose of 2 × 10^10^ vg/mouse). Untreated SCID/mdx and C57BL/6 mice were used as controls. Results were presented as mean ± standard error of the mean, *p < 0.05; ***p < 0.001, and ****p < 0.0001 using Student’s *t*-test (*n* = 4–5)

SCID/mdx mice treated with the ssAAV9-Sk-CRM4-Des-MD1Δ and ssAAV9-Sk-CRM4-Des-FST alone or in combination, outperformed the untreated control mice in treadmill test 16 weeks post vector injection (Fig. 6a). In particular, the performance of dystrophic mice treated with the ssAAV9-Sk-CRM4-Des-MD1Δ or ssAAV9-Sk-CRM4-Des-FST vector increased with 300% (*t*-test; *p* < 0.001) and 250%, respectively, compared to untreated dystrophic mice. Most importantly, the performance of dystrophic mice that received combination gene therapy by co-delivery of both ssAAV9-Sk-CRM4-Des-MD1Δ and ssAAV9-Sk-CRM4-Des-FST showed a robust 356% increase, compared to untreated dystrophic mice (Fig. 6a). This was consistent with a significant 1.8-fold increase (*t*-test; *p* < 0.05) in absolute tetanic contraction force ex vivo on isolated extensor digitorum longus (EDL) muscle from SCID/mdx mice that received the combination gene therapy, approximating the EDL absolute contraction force in wild-type C57BL/6 mice (191 + 58 mN in MD1Δ/FST-treated SCID/mdx; 105 + 13 mN in untreated SCID/mdx; 232 + 42 mN wild-type C57BL/6).

We subsequently corroborated the functional correction after gene therapy by histopathological examination of the skeletal muscle. The central localization of the nucleus in the muscle fibers is one of the hallmarks of muscular dystrophy, as opposed to peripheral nuclear localization in normal, non-dystrophic muscle fibers. Hematoxylin and eosin staining of the tibialis anterior muscle revealed a significant reduction (*t*-test; *p* < 0.0001) in centrally nucleated muscle fibers after systemic gene therapy with ssAAV9-Sk-CRM4-Des-MD1Δ and/or ssAAV9-Sk-CRM4-Des-FST compared to non-treated SCID/mdx mice (Fig. 6b, c). Moreover, the combination therapy based on co-delivery of both ssAAV9-Sk-CRM4-Des-MD1Δ and ssAAV9-Sk-CRM4-Des-FST resulted in an even more pronounced and significant effect (*t*-test; *p* < 0.001) on the nuclear re-localization towards the muscle fiber periphery than by either of the single vector treatments (Fig. 6b, c). Furthermore, the fiber cross-sectional area also increased after gene therapy with the Sk-CRM4-based vectors. In particular, treatment of SCID/mdx mice with the ssAAV9-Sk-CRM4-Des-MD1Δ vector significantly increased the cross-sectional area 130% (*t*-test; *p* < 0.001) (Fig. 6d) compared to untreated SCID/mdx mice. The effect was even 250% greater following ssAAV9-Sk-CRM4-Des-FST treatment (*t*-test; *p* < 0.0001), exceeding the fiber cross-sectional area typically observed in wild-type mice (Fig. 6d). The maximum effect could be attained upon co-delivery of ssAAV9-Sk-CRM4-Des-MD1Δ and/or ssAAV9-Sk-CRM4-Des-FST resulting in a 285% increase in fiber cross-sectional area (*t*-test; *p* < 0.001). In conclusion, the impact of gene therapy on the improvement of the histopathological features characteristic of a dystrophic phenotype was consistent with its effect on overall physical performance and muscle function.

### Comparative analysis of therapeutic efficiency

**Fig. 7:**
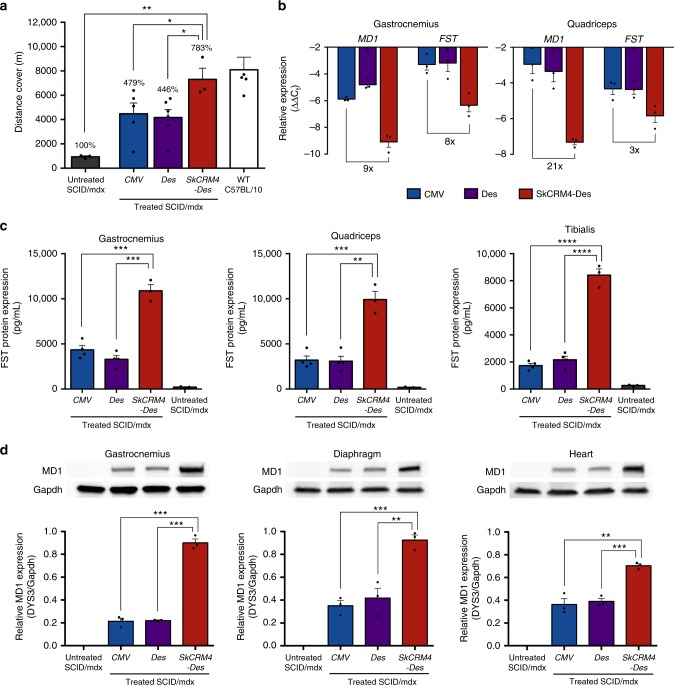
Comparison of *Sk-CRM4/Des* chimeric promoter with conventional promoters in SCID/mdx. The ssAAV9 vectors encoding *MD1* and *FST* under the control of the *Sk-CRM4/Des* chimeric promoter, *Des*, or *CMV* conventional promoters were co-injected into SCID/mdx mice at a dose of 1 × 10^11 ^vg/mouse for each vector. **a** Physical performance of the 16 weeks post-injected SCID/mdx mice treated with the different therapeutic vectors compared to the untreated age-matched control SCID/mdx and wild-type C57BL/10ScSnJ mice were determined by measuring the distance covered. Results were presented as mean ± standard error of the mean (*n* = 3–6). The percentages on the top indicate the distance related to untreated SCID/mdx mice. **b** Human *MD1* and *FST* mRNA quantification in skeletal muscles (i.e., gastrocnemius and quadriceps) of treated SCID/mdx mice, expressed relative to the housekeeping gene Gapdh by qRT-PCR. The Gapdh protein levels were used as loading control for normalization. **c** Quantification of human FST protein (in pg/50 mg of skeletal muscle tissues) as determined by ELISA. **d** Western blot analysis of MD1 protein expression in heart, diaphragm and skeletal muscle (*gastrocnemius*) of treated and control SCID/mdx. The protein expression levels of MD1 were detected using a dystrophin-specific (DYS3) antibody. Statistical analysis using *t*-test *p < 0.05; **p < 0.01; ***p < 0.001, ****p < 0.0001; mean + s.e.m. (*n* = 3–6)

The performance of the *Sk-CRM4/Des* chimeric promoter was subsequently compared with other conventional promoters, such as *Des* and *CMV* (Fig. 1i, j, l, m), which are generally used for therapeutic gene expression in skeletal muscles and being used in clinical trials for muscle-directed gene therapy. Comparative functional analysis 16 weeks post-injection (Fig. 7a) revealed that robust and sustained phenotypic correction could be achieved in SCID/mdx mice co-injected with the ssAAV9-SkCRM4-Des-MD1 (Fig. 1i) and ssAAV9-SkCRM4-Des-FST (Fig. 1l) vectors resulting in a normalization of the running distance covered, comparable to that of wild-type mice (Student’s two-tailed *t*-test; *p*-value = 0.6245, non-significant). This represent a robust ≈800% improvement in distance traveled compared to the untreated dystrophic SCID/mdx mice (*t*-test; *p* < 0.01). Hence, this is consistent with a bona fide cure of muscular dystrophy in this preclinical model when the next-generation AAV9 vectors that expressed the *MD1* and *FST* genes from the de novo designed *SkCRM4-Des* promoter. Most importantly, the therapeutic effect was significantly increased (*t*-test; *p* < 0.05) when the *SkCRM4/Des* promoter was used compared to when the therapeutic *MD1* and *FST* genes were driven from either *CMV* or *Des* promoters (Fig. 7a).

**Fig. 8:**
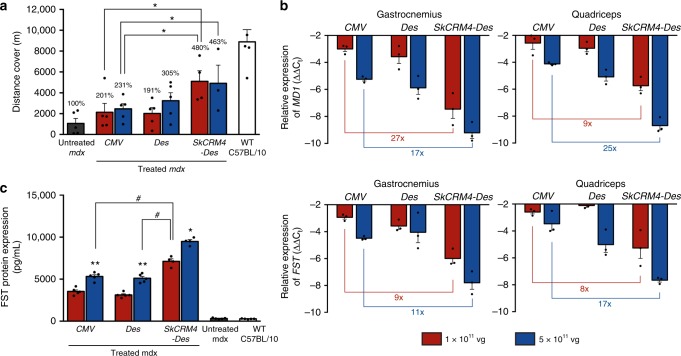
Comparison of *Sk-CRM4/Des* chimeric promoter with conventional promoters in mdx mice model. The ssAAV9 vectors encoding *MD1* and *FST* gene under *Sk-CRM4/Des* chimeric promoter, *Des*, or *CMV* were co-injected as a combination therapy into mdx mice at a dose of 1 × 10^11^ vg/mouse or 5 × 10^11^ vg/mouse. **a** Treadmill test of the 20-week-old mdx mice (16 weeks post-injection) treated with the different constructs of therapeutic vectors compared to the untreated mdx and wild-type C57BL/10ScSnJ mice. The results were shown as the distance covered. The percentages indicate the distance related to untreated mdx mice. Statistical analysis using *t*-test *p < 0.05 (*n* = 4–5). **b** Human *MD1* and *FST* mRNA quantification in skeletal muscles, i.e., gastrocnemius and quadriceps of treated mdx mice, expressed relative to the housekeeping gene *Gapdh* by qRT-PCR. **c** Quantification of human FST protein as measured using ELISA. Results were presented as mean ± standard error of the mean. Statistical analysis using *t*-test *p < 0.05, **p < 0.01 (related to 1 × 10^11^ injected group of the same construct), #*p* < 0.05; mean + s.e.m. (*n* = 4–5)

Next, the *MD1* and *FST* mRNA and protein expression levels were determined using qRT-PCR (Fig. 7b) and ELISA (Fig. 7c) or western blotting (Fig. 7d), respectively. The new muscle-specific *Sk-CRM4/Des* chimeric promoter led to a robust increase in *MD1* and *FST* (Figs. 7b and  8b) mRNA expression levels compared to when *CMV* or *Des* promoters were used, as control. This translated in a significant (*t*-test; *p* < 0.01) increase in MD1 in heart, diaphragm and skeletal muscle (*gastrocnemius)* (Fig. 7d) and FST (Fig. 7c) protein expression when the *Sk-CRM4/Des* chimeric promoter was used compared to the *CMV* or *Des* promoters. The western blot analysis confirmed that MD1 had the expected molecular weight (136 kD) (Fig. 7d and Supplementary Fig. 6). Hence, the increased therapeutic efficacy with the next-generation AAV vectors expressing the MD1 and FST therapeutic genes from the *Sk-CRM4/Des* chimeric promoter was consistent with the increase in MD1 and FST expression. These results suggested that the use of the *Sk-CRM4/Des* chimeric promoter provided an effective and sustained therapeutic effect that is significantly more robust compared to conventional promoters typically used for DMD gene therapy.

In addition to the SCID/mdx model, the efficacy of the *Sk-CRM4/Des* constructs for DMD treatment was also validated in an immunocompetent dystrophic mouse model (mdx) and compared with conventional promoters (Fig. 8a–c). Co-injection of mdx mice with the ssAAV9-SkCRM4-Des-MD1 and ssAAV9-SkCRM4-Des-FST vectors resulted in a significant increase (*t*-test; *p* < 0.05) in therapeutic efficacy based on a treadmill assay, at all vector doses tested, consistent with a significant increase in *MD1* or *FST* mRNA (Fig. 8b) or protein expression levels (Fig. 8c). Notably, even at a fivefold lower vector dose (i.e., 10^11^ vg) the *SkCRM4-Des*-containing vectors outperformed the vectors that expressed the *MD1* or *FST* therapeutic transgenes from either the *CMV* or *Des* promoters resulting in higher transgene expression levels resulting in a significant increase in overall therapeutic efficacy. Collectively, these results indicate that this new *SkCRM4* transcriptional element lowers vector dose requirement.

Lastly, we examined the extent of the apoptosis using a TUNEL assay. Analysis of skeletal muscle (tibialis anterior) from mdx mice injected with the different AAV9 vectors expressing MD1 or FST from the *SkCRM4-Des, Des* or *CMV* promoters (at 5 × 10^11^ vg/mouse for each vector) revealed that there was no significant increase in apoptotic cells compared to untreated control mdx or wild-type control mice (Supplementary Fig. 7a). Moreover, immunohistochemistry for staining CD4 and CD8-positive T cells in the muscles revealed no significant increase in T-cell infiltration in skeletal muscle from mdx mice injected with the various AAV9 vectors expressing MD1 or FST from the *SkCRM4-Des, Des* or *CMV* promoters (Supplementary Fig. 7b). Hence, this indicates that over-expression of MD1 or FST in the skeletal muscle with the next-generation ssAAV9-SkCRM4-Des-MD1 and ssAAV9-SkCRM4-Des-FST vectors was considered to be relatively safe.

## Discussion

In this study, we have identified novel and potent muscle-specific *CRMs* using an improved genome-wide computational algorithm. In particular, the most potent *Sk-CRM4* element resulted in a substantial improvement in transcription (up to 400-fold) when combined with the muscle-specific desmin promoter, one of the most commonly used muscle-specific promoters^35,39,40^. In addition, a 36-fold increase in cardiac expression could be attained. To our knowledge, this type of computational vector design represents the first of its kind to improve the performance of a gene therapy vector for muscle-directed gene therapy, yielding an unprecedented increase in muscle-specific gene expression. Indeed, our previously identified cardiac-specific *CRM* failed to increase muscle-specific expression from the synthetic *SPc5-12* promoter, in contrast to the present study^41^. Moreover, the muscle-specific *Sk-CRM4/Des* chimeric promoter outperformed the commonly used *CMV* promoter, up to 100- to 200-fold in most muscle groups and thus provides an attractive alternative^34,42^. In particular, the *CMV* promoter was employed to drive expression of the lipoprotein lipase (LPL^S447X^) gene that constitutes the basis of the first approved AAV-based gene therapy product^6,7^. Our current results underscore the potential of these computationally designed *CRMs* to improve the efficacy of skeletal muscle-directed and cardiac gene therapy of DMD, with broad implications for other muscle disorders. In particular, the combination of the de novo designed *Sk-CRM/Des* muscle-specific promoter with the use of the AAV9 muscle-tropic serotype resulted in widespread expression of reporter or therapeutic genes (i.e. micro-dystrophin, follistatin) in heart and skeletal muscle following systemic gene delivery in neonatal or adult mice. This was consistent with robust phenotypic correction of the dystrophic phenotype in DMD mice resulting in long-term improvement of the physical performance and restoration of several characteristic dystrophic pathophysiological features, particularly increased muscle fiber size and re-localization of the fiber nuclei from the nuclei towards the periphery. Previous efforts to augment muscle-directed gene expression by promoter optimization and/or AAV capsid engineering typically resulted in more modest and incremental increases in gene expression. Moreover, though muscle-specific promoters are often preferred by virtue of their tissue-specificity, they typically yield much lower expression levels than ubiquitously expressed promoters like *CMV*^35^ that are sometimes prone to transcriptional silencing in vivo^43^. The incorporation of the *Sk-CRM4* element upstream of the desmin (*Des*) promoter overcomes this limitation by boosting muscle-specific gene expression levels by several orders of magnitude, based on reporter genes (i.e., luciferase). The generation of synthetic muscle-specific promoters (i.e*.*, *SPc5-12*) by molecular assembly and selection of muscle-specific *TFBS* combinations in muscle cell lines in vitro has been proposed as an alternative strategy to boost muscle-specific gene expression^31,44^. However, this in vitro selection approach resulted in a relatively modest and incremental six-fold increase in muscle-specific expression relative to the *CMV* promoter, which is in contrast to the robust 100-fold increased expression attained with the computationally designed *Sk-CRM4/Des* chimeric promoter relative to *CMV*. In particular, head-to-head comparative analysis revealed that the *Sk-CRM4/Des* promoter is 15- to 30-fold more potent than the synthetic *SPc5-12* promoter (Fig. 4a–d). The current computational approach distinguishes itself from this synthetic molecular assembly and in vitro selection method by allowing comprehensive and genome-wide identification of evolutionary conserved *TFBS* clusters that are associated with highly expressed, muscle-specific genes in human muscle in vivo. Consequently, this translated into a more robust increase in expression after gene therapy in vivo, compared to what could be achieved using synthetic promoters obtained by in vitro assembly and selection^31,44^.

The different muscle-specific human *CRMs* were retrieved from their natural in vivo context following a genome-wide screening and contained a molecular signature composed of *TFBS* clusters (containing *TFBS* for E2A, CEBP, LRF, MyoD, SREBP, Tal1, PPAR) that are characteristic of highly expressed genes in the muscle. In particular, the *Sk-CRM4* and *Sk-CRM1* elements are among the most potent muscle-specific *CRMs* that have several *TFBS* in common (i.e., *E2A, LRF, CEBP*, and *MyoD*). They were derived from quintessential human muscle-specific genes *MYLPF* and *APTP2A1* that encode for the myosin light chain and the sarcoplasmic/endoplasmic CA^2+^ ATPase (SERCA), respectively. Since *MYLPF* and *APTP2A1* are typically highly expressed in fast twitch muscle, this may possibly account for the higher activity of *Sk-CRM4* and *Sk-CRM1* in the fast twitch skeletal muscles, whereas their activity was lower in low twitch muscle, like diaphragm. Nevertheless, the reduced gene transfer in the diaphragm compared to that in other muscles may also have contributed to the reduced gene expression (Supplementary Fig. 2). The increased protein expression from the *CRM*-driven constructs correlated strongly with increased transcriptional activity. This was consistent with the increased binding of CEBP and SRF on the *MYLPF* promoter regions encompassing the respective TFBS that were mapped within the Sk-CRM4 element.

The main advantage of this novel computational approach used to identify the tissue-specific *CRMs* is that it takes into account the actual context-dependent *TFBS* interactions from a broad genome-wide perspective in vivo, instead of just relying on the over-representation of a single *TFBS* in a given tissue-specific *CRM*. Consequently, this computational analysis is more comprehensive and allows for the identification of *TFBS* elements that tend to cluster together in *CRMs* of genes that are highly expressed in the skeletal muscle and the heart (Table 1), based on a differential distance matrix-multidimensional scaling (DDM-MDS)^45^. DDM-MDS is a count-based method that evaluates the statistical significance of the observed versus expected numbers of TFBSs in the sets of promoters. The main difference with methods that only consider over-representation is that a data structure is used that allows simultaneous evaluation of the degree of association between TFBSs for different TFs. The DDM-MDS method^45^ produces more relevant and/or reproducible results compared to other methods such as LogicMotif^46^, POCO^47^, and CRÈME^48^. Another advantage of this DDM-MDS approach is does not need to rely on a comprehensive database containing all muscle-specific genes, but merely a gene set corresponding to the most highly expressed genes, The analysis is most robust if we restrict ourselves to a selection of these most highly expressed muscle-specific genes rather than a comprehensive list of all muscle-specific genes (see Supplementary Method). In the current study, the analysis was based on 29 promoters which falls in a similar range as our recent studies, that are based on 43 or 59 to identify cardiac-specific or liver-specific CRMs, respectively^41,49,50^.

Our computational approach takes into account cross-species evolutionary conservation of the selected *Sk-CRM* elements (Supplementary Fig. 1). Cross-species comparison significantly improves genome-wide prediction of *CRMs*^51,52^. This justifies prioritizing *CRMs* based on evolutionary conservation. Consequently, such evolutionary conserved *CRMs* are likely to exhibit the same properties across distinct species with respect to tissue-specificity and expression levels. This is important for gene therapy as tissue-specificity and expression levels based on a given *CRM* may then likely be conserved in preclinical models and human subjects. This potentially improves the prospects for clinical translation. Indeed, if a sequence is conserved and is associated with highly expressed genes in a given species, it will likely be associated with high expression in other species as well. Nevertheless, evolutionary conservation in as of itself does not necessarily imply that the *CRMs* are evolutionarily optimized and selected for to maximize expression.

Open chromatin structures, as defined in the ENCODE database^53^ were considered in the current computational analysis, which distinguishes the current approach from our previous strategies used to maximize tissue-specific gene expression after gene therapy^41,49,50^. We, therefore, considered chromatin contextual features better describing features like DNA accessibility, nucleosome occupancy, or the presence of specific histone post-translational modifications associated with transcription regulation. In practice, candidate *Sk-CRMs* were mapped against the human hg19 genome using the blat tool at the UCSC genome browser (https://genome.ucsc.edu). Next, the mapped regions were visualized along the ENCODE Regulation supertrack containing information relevant to regulation of transcription from the ENCODE project. The layered H3K4Me1 and layered H3K27Ac marks are epigenetic signatures often found near regulatory elements, while the DNase I hypersensitivity tracks indicate where chromatin is hypersensitive to cutting by DNase I and are associated with both regulatory regions and promoters. The open chromatin structure is used as a filter in the selection of candidate *CRMs* because this indicates that, at least in some conditions, a particular genomic region is accessible to TFs. In contrast, some chromatin features may reduce the accessibility of the DNA and therefore impede expression, despite the presence of other favorable features. Moreover, chromatin effects are known to influence expression from AAV vectors as AAV adopts a chromatin-like configuration^54–56^.

Enhancers typically activate gene expression when they are located at far greater distances from the transcriptional start site (*TSS*). However, such distal enhancers may increase the risk of insertional oncogenesis following vector integration in the target cell genome^57,58^. Hence, we strategically focused on selecting *CRMs* that are located within the proximal promoter (i.e., < 2.5 kb from *TSS*) to diminish the risk of insertional oncogenesis when used in the context of gene therapy. We have previously demonstrated that *CRMs* that have been identified with the DDM-MDS method and that are located within a 2 kb region from the TSS (i.e., HS-CRM8^49,50^), do not significantly increase the risk of insertional oncogenesis, in normal or highly sensitive tumor-prone mouse models, even when vectors are employed that integrate at high efficiencies in the target cell genome^59^.

The overall efficiency of this next-generation AAV-based muscle-directed gene therapy compares favorably to the state of the art vectors as higher expression levels could be attained leading to an increased therapeutic efficacy. Previous studies were based on intramuscular AAV injections, which did not allow body-wide expression of the therapeutic micro-dystrophin and consequently resulted in a more modest correction of the dystrophic phenotype^30,60^. Moreover, substantial correction of the dystrophic phenotype could be achieved in a physical endurance test (i.e., treadmill assay) either using a single or a combination of therapeutic genes (i.e., *MD1* or *FST*).

The overall impact of the *Sk-CRM4* element on gene expression may vary depending on the transgene. Indeed, though the *Sk-CRM4* element led to a significant increase in both *MD1* and *FST* transcription, the highest relative- increase in mRNA levels was apparent when luciferase was used. Moreover, the significant increase in MD1 and FST protein levels was not directly proportionate to the increased transcription. This suggests that additional, potentially unknown, post-transcriptional or translational bottlenecks may influence gene therapy efficacy. Though over-expressing a potential therapeutic gene might lead to imbalanced protein synthesis followed by muscle fiber death, lack of apoptosis or T-cell infiltration further supports the safety of the current vector designs and allowed for the use of lower vector doses to attain an increased therapeutic effect compared to more conventional vector designs.

Other studies that were based on systemic intravenous injection of AAV vectors encoding therapeutic genes typically required higher vector doses than the therapeutic doses used in the present study^16,17^ and/or requiring the use of vascular endothelial growth factor (VEGF^164^), raising additional safety concerns. Lower vector doses may minimize the risk of potential AAV-specific T-cell immune response in human trials and may ease manufacturing constraints. The first gene therapy trials for DMD had been initiated based on intramuscular delivery of an AAV2.5 variant encoding mini-dystrophin^20^. Overall, dystrophin-expression in patient’s biopsies was either absent or limited. Similarly, the use of oligonucleotide-based exon-skipping approaches failed to demonstrate any long-term effects^25,61^. The next-generation vector design described in the present study may potentially overcome some of these bottlenecks and pave the way towards clinical applications to treat DMD and other muscle disorders.

## Methods

### Identification of *Sk-CRM*

We designed a computational approach (Fig. 1a) (Supplementary Methods) to specifically identify robust skeletal muscle *CRMs* (*Sk-CRMs)*. This approach consisted of five steps: (1) muscle-specific genes were identified that are highly and lowly expressed based on statistical analysis of micro-array expression data of normal human tissues;^62,63^ (2) publicly available databases were used to extract the corresponding promoter sequences (NCBI36/hg18 genome assembly) (3) a differential distance matrix (DDM)-multidimensional scaling (MDS) approach^45^ was used to identify the regulatory modules and the *TFBS* they contain (4) the genomic context of the highly expressed genes was then screened for evolutionary conserved clusters of *TFBS* (i.e., *CRMs*). A detailed description of these four successive steps for identification of tissue-specific *CRM* was described, that led to the identification of robust liver-specific or cardiac-specific *CRMs*^41,49,50^. However, in the present study we further refined this computational analysis by adding an extra step that also takes into consideration biochemical features associated with transcription regulation and/or open chromatin structures, as defined in the ENCODE database (Supplementary Methods)^53^. Six different *Sk-CRMs* were selected based on this computational analysis (Table 1).

### Generation of *Sk-CRMs* AAV constructs

The *CRMs* were synthesized by conventional oligonucleotide synthesis (Geneart, Thermo Fisher Scientific, Carlsbad, California, USA) and were flanked with *MluI* and *Acc65I* restriction sites at the 5ʹ and 3ʹ’ ends, respectively. After synthesis, the *Sk-CRMs* were cloned upstream of the desmin (*Des*) promoter (983 bp) (Invivogen, France) in a self-complementary adeno-associated viral (*scAAV*) vector^64^ (kindly provided by Dr. Srivastava, University of Florida College of Medicine, USA). This vector encoded for the firefly luciferase (*Luc*) reporter gene and also contained the Minute Virus of Mouse (*MVM*) intron and a simian virus 40 (SV40) polyadenylation site (*pA*). The corresponding scAAV constructs that contained these *Sk-CRM* elements were designated as pscAAV-Sk-CRM-Des-Luc (Fig. 1c). An AAV vector without the *Sk-CRM* was used as control (designated as pscAAV-Des-Luc*)* (Fig. 1b). For comparison, the most robust *Sk-CRM4* (Supplementary Table 1) was cloned in combination with a synthetic promoter *(SPc5-12)*^31^, known for expression in the skeletal muscle and heart, within the scAAV vector backbone to drive the luciferase reporter (designated as pscAAV-Sk-CRM4-SPc5-12-Luc) (Fig. 1f). The corresponding control AAV vector without the *Sk-CRM4* was also generated (designated as pscAAV-SPc5-12-Luc*)* (Fig. 1e). Additionally, the cytomegalovirus promoter (*CMV*)^17^, often used as gold standard to achieve high expression in the muscle, was cloned into the *scAAV* backbone encoding luciferase for comparison (designated as pAAV-CMV-Luc-SV40pA) (Fig. 1d). Next, five different sub-fragments of *Sk-CRM4* were synthesized by conventional oligonucleotide synthesis and were cloned upstream of the desmin promoter in the same *scAAV* backbone encoding luciferase. The constructs generated were designated as pssAAV-*Sk-CRM*^(a-e)^-*Des-Luc*. In order to validate these *CRMs* for gene therapy of DMD, we cloned human micro-dystrophin 1 (*MD1*)^30^ or an alternative truncated version (*MD1Δ*) containing additional deletions of the spectrin-like repeats (Supplementary Fig. 5) and human follistatin (*FST344*)^34^ cDNA into a single-stranded AAV (ssAAV) vector driven from the *Sk-CRM4/Des* chimeric promoter. The *MD1* cDNA was flanked by *MluI* and *XhoI* restriction sites at the 5ʹ and 3ʹ ends while the *FST* cDNA was flanked by *MluI* and *SalI* restriction sites at the 5ʹ and 3ʹ, respectively, and were synthesized by conventional oligonucleotide synthesis. The *Sk-CRM4/Des* was cloned upstream of the *MVM* intron to drive the expression of the *MD1* gene or *FST* gene in the context of a single-stranded adeno-associated viral vector (ssAAV) backbone that also contained a 49 bp synthetic polyadenylation site^65^. The constructs were designated as pssAAV-Sk-CRM4-Des-MD1 (Fig. 1h) and pssAAV-Sk-CRM4-Des-FST (Fig. 1k), respectively. In this vector, the *FST* cDNA was linked to a *Luc* reporter gene via a 2A polypeptide-encoding fragment. Cloning details are available upon request.

### AAV vector production and purification

AAV vectors were prepared as described in the Supplementary Methods^36^. Genome-containing vectors and empty AAV capsid particles were purified by cesium chloride gradient. The vector titer (in vector genomes or vg/ml) for all vectors was determined by quantitative (q)PCR (Supplementary Methods). Typically, vector titers achieved for all vectors were in the range of 5 × 10^11^–2 × 10^12^ vg/ml.

### Mice

This study was carried out in CB17/IcrTac/Prkdc^scid^ mice (Taconic, Denmark), C57BL/6 mice (Janvier Labs, France) or SCID/mdx mice (kindly provided by Dr. Y. Torrente and Mrs. M. Meregalli, University of Milan, Italy). The dystrophic mdx and CB57BL/10ScSnj mice models were purchased from Jackson Laboratory, USA. All animal procedures were carried out at the Vrije Universiteit Brussel and the University of Leuven (Belgium) and were approved by the respective Institutional Animal Ethics Committees (i.e., KU Leuven & VUB Ethische Commissie Dieren—ECD). Mice were housed under specific pathogen-free (SPF)-like conditions; food and water were provided ad libitum. For each cohorts of various experiments, mice were randomly selected from a group of mice of same age and similar weight to standardize control and experimental groups as much as possible. Mice and samples were coded based on the ear tag number. The ear tag number was decoded after conducting the experiment. The designated investigator injecting the vectors was blinded to the experimental outcome. The investigators were blinded to the precise nature of the CRMs or vectors.

### In vivo bioluminescence analysis

For the in vivo screening of the AAV vectors containing the different *Sk-CRMs* and the *Luc* reporter gene, 2-days old CB17/IcrTac/Prkdc^scid^ mice were intravenously injected into the retro-orbital vein with 50 μl of purified AAV9 vectors (5 × 10^9^ vg/mouse). The experimental mice were subjected to bioluminescence imaging analysis at 5 and 6 weeks post-injection using an in vivo optical imaging system (PhotonImager, Biospace Lab, Paris, France). Alternatively, AAV9 vectors were injected intravenously into the tail vein into adult 6-weeks-old CB17/IcrTac/Prkdc^scid^ adult mice at a dose of 1 × 10^10^ vg per mouse and analyzed by bioluminescence imaging 2 and 4 weeks post-injection. Before imaging, mice were intravenously administered with a d-luciferin substrate (30 mg/ml) at a dose of 150 mg/kg of body weight. Quantitative image analysis of individual organs was performed at 7 weeks post vector injection for the mice that were injected as neonates and at 6 weeks post vector injection for the injected adult cohorts. In this case, mice were euthanized by cervical dislocation within 1-minute post d-luciferin administration. In vivo bioluminescence was expressed in photons (ph) s^-1^ cm^-2^ steradian (sr)^-1^ and displayed as a pseudo-color overlay onto a gray scale animal image using a rainbow color scale. All images were scaled to the same maximum and minimum, as represented in the color bar.

### Transduction efficiency, biodistribution, and mRNA levels

Transduction efficiency and biodistribution was evaluated by quantifying *Luc* transgene copy numbers in the different organs and tissues, as described in Supplementary Methods. The qPCR results were expressed as mean AAV copy number/100 ng of genomic DNA. To quantify *Luc* mRNA levels in different tissues real-time qPCR analysis was performed using *Luc*-specific primers (amplicon 206 bp) (Supplementary Methods). All results were normalized to mRNA levels of the endogenous murine glyceraldehyde-3-phosphate dehydrogenase (*Gapdh*) gene (amplicon 82 bp).

### Chromatin immunoprecipitation assay (ChIP assay)

To verify the biding of selected TF on the cognate *TFBS*, ChIP assays were performed (Supplementary Methods) ^67^. Genomic DNA regions of interest were isolated using 4 μg of SRF-specific antibody (sc-335; Santa Cruz Biotechnology, Santa Cruz, CA, USA) or a CEBP-specific antibody (sc-150; Santa Cruz Biotechnology, Santa Cruz, CA, USA). qPCR reactions were carried out in triplicate on specific genomic regions using SYBR Green Supermix (Bio-Rad, USA). The resulting signals were normalized for primer efficiency by carrying out qPCR for each primer pair using the genomic input DNA with *Sk-CRM4-*specific forward and reverse primers. Negative control primers were purchased from Active Motif (Carlsbad, CA, USA) (#71012) and are specific for non-transcribed gene sequences on chromosome 17.

### Analysis of therapeutic efficacy in vivo

Four-weeks-old SCID/mdx or mdx mice were injected intravenously into the tail vein with the indicated doses of the ssAAV9-Sk-CRM4-Des-MD1 (or ssAAV9-Sk-CRM4-Des-DM1Δ) and/or ssAAV9-Sk-CRM4-Des-FST therapeutic vector. Control mice were injected with phosphate buffered saline (PBS). Sixteen weeks post-injection, the treated and control SCID/mdx or mdx mice and wild-type age-matched C57BL/6 or C57BL/10ScSnJ mice were subjected to a treadmill test (Exer-3/6 open treadmill, Columbus Instruments, USA) to determine correction of the dystrophic phenotype. The inclination of the belt was adjusted to 10° uphill before performing the test. The initial speed was set at 10 m min^-1^ and thereafter the speed was increased by 1 m min^-1^ every minute. The test was terminated at a point when the mice sat for ≥ 5 s on the pulse grill. At that point the distance covered by the mice was recorded and the total distance covered by the mice during the course of the test was calculated by using the formula, according to the manufacturer’s instructions: distance (in m) = (*N* + *n*)/2)*(*N* − *n* + 1*)* where *N* is the speed (in m min^-1^) at the point of termination of the test and n is the speed (in m min^-1^) at the start point of the test.

### In vitro analysis of muscle contractile properties

Mice injected with the ssAAV9-Sk-CRM4-Des-MD1Δ and ssAAV9-Sk-CRM4-Des-FST vectors (2 × 10^10^ vg per mouse) were euthanized 18 weeks post vector administration and the extensor digitorum longus muscle was isolated from each mouse to perform the force test. Age-matched C57BL/6 mice and untreated SCID/mdx mice were used as controls. Functional analysis was performed using an Aurora 1200 A in vitro muscle test system, as previously reported^67^. Briefly, freshly isolated muscles were electrically stimulated in a temperature-controlled (30° C) chamber, filled with Krebs-Ringer bicarbonate buffer solution containing 10 mM glucose and continuously gassed with 99% O_2_. The electrical stimulation was performed by means of a couple of platinum electrodes, located 4 mm from each side of the muscle. Tetanic force was evoked by stimulation with a train of 0.2 ms pulses (150 Hz pulses for 0.5 s).

### Histological analysis

Mice injected with the ssAAV9-Sk-CRM4-Des-MD1Δ and ssAAV9-Sk-CRM4-Des-FST vectors (2 × 10^10^ vg per mouse) were euthanized 18 weeks post vector injection and the tibialis anterior muscles were harvested for hematoxylin and eosin staining and (Supplementary Methods). For immunofluorescence staining, the heart and the tibialis anterior muscles were subjected to cryofixation and stained for mouse laminin and human dystrophin using specific antibodies and the appropriate fluorescein-isothyocyanate (FITC) or tetramethylrhodamineisothiocyanate-conjugated (TRITC) anti-mouse or anti-rabbit (1:500, Life Technologies, Carlsbad, CA, USA) (Supplementary Methods). Nuclear staining was performed using 4’,6-diamidino-2-phenylindole (DAPI; Hoechst nucleic acid stain, Life technologies, USA) (1:1000 dilution) for 1 h at room temperature. For assessment of the immune response, infiltration of CD4^+^ and CD8^+^ T-cells was analyzed. CD4 and CD8-specific antibodies (1:500; Cell signaling, USA) were used as primary antibody to detect mouse T cells. After washing, the samples were subjected to HRP-conjugated secondary antibody (1:2000; Abcam, UK). Substrate was added (3,3’-diaminobenzidine tetrahydrochloride) (DAB kit, Cell signaling, USA) and tissue sections were counterstained using hematoxylin. Spleen sections were used as control. Cellular apoptosis was assessed in situ based on a TUNEL assay. Modified dUTPs were, therefore, incorporated by the enzyme terminal deoxynucleotidyl transferase (TdT) at the 3’-OH ends of fragmented DNA using Click-iT® TUNEL Alexa Fluor647® Imaging Assay (Life Technologies, Carlsbad, CA, USA), according to the manufacturing protocol. Tissue samples were then analyzed under a fluorescent microscope (Nikon Eclipse 80i) and images were processed by NIS Elements 4.30.02 software.

### FST enzyme-linked immunosorbent assay

Human FST was quantified from the muscles of SCID/mdx mice by an enzyme-linked immunosorbent assay (ELISA) with a human FST-specific immunoassay kit (Quantikine; R&D Systems, Minneapolis, USA), according to the manufacturer’s protocol. Briefly, total soluble protein was extracted from 50 mg gastrocnemius, quadriceps, and/or tibialis anterior muscle with CelLytic MT Mammalian Tissue Lysis reagent (Sigma, St. Louis, Missouri, USA). A total of 100 μl of lysate was loaded per well, and muscle FST concentrations were determined against a standard curve made with recombinant human FST provided by the manufacturer.

### Western blotting

Proteins were extracted from skeletal muscles, diaphragm, or heart by solubilization in lysis buffer (50 mM Tris, 5 mM EDTA, 5% sodium dodecyl sulfate, 5% glycerol) supplied with 1x protease inhibitor cocktails (Thermo Fisher Scientific, USA). The total protein concentrations were determined using RC DC™ Protein Assay (Bio-Rad, USA), per manufacturer’s protocol. Then 50–100 microgram of proteins were mixed with sample buffer (Bio-Rad, USA) and loaded into 4–15% TGX gels (Bio-rad, USA) for sodium dodecyl sulfate-polyacrylamide gel electrophoresis. The proteins were transferred to 0.45 μm polyvinylidene fluoride membranes (Invitrogen, USA) using Towbin buffer (Bio-rad, USA). Membranes were blocked with 3% blotting-grade blocker (Bio-rad, USA) for 1 h at room temperature following by incubation with 1:100 mouse anti-human dystrophin antibody (NCL-DYS3; Novocatsra, UK) or 1:1000 rabbit anti-GAPDH antibody (#2118; Cell Signaling Technology, Netherland) in 3% Blotting-grade blocker. After washing three times with 1x TBS-T buffer, the secondary antibodies compatible with primary antibody, e.g., horseradish peroxidase (HRP)–conjugated 1:2000 goat anti-mouse (#7076; Cell Signaling Technology, Netherland) and 1:10,000 goat anti-rabbit IgG (#7074; Cell Signaling Technology, Netherland) were incubated with the membranes for 1 h at room temperature. Afterward, membranes were washed three times for 5 min each with TBS-T buffer. The chemiluminescent signal was developed using the SuperSignal West Pico PLUS substrate (Thermo Fisher Scientific, USA). The sizes of protein were estimated using MagicMarker XP Protein Standard (LC5602; Invitrogen, USA) (Supplementary Fig. 6). The signals were quantified under Image-J software.

### Statistics

Data were analyzed using Microsoft Excel Statistics package or Prism Graphpad. Values shown in the figures are the mean + s.e.m. Specific values were obtained by comparison using *t*-test or one-way ANOVA. Samples were tested (*n* = 3–5, twice) and representative results are shown.

### Reporting summary

Further information on experimental design is available in the Nature Research Reporting Summary linked to this article.

## Supplementary information

Supplementary Information

Reporting Summary

## Data Availability

The authors declare that all data supporting the findings of this investigation are available within the article, its Supplementary Information, and from the corresponding authors, upon reasonable request. All comprehensive western blot images including the molecular weight markers were presented in Supplementary Fig. 6.
