# Supplementary material for: Next-generation muscle-directed gene therapy by in silico vector design
Source: Nat Commun. 2019 Jan 30;10:492. doi: 10.1038/s41467-018-08283-7 (PMC6353880; doi:10.1038/s41467-018-08283-7)
Supplement: Supplementary file 1 — Supplementary Information [file 41467_2018_8283_MOESM1_ESM.pdf]

Supplementary Information

NEXT-GENERATION MUSCLE-DIRECTED GENE THERAPY BY *IN SILICO* VECTOR DESIGN

Sarcar *et al.*

Supplementary Figure 1

a Sk-CRM1 (*ATP2A1* gene)

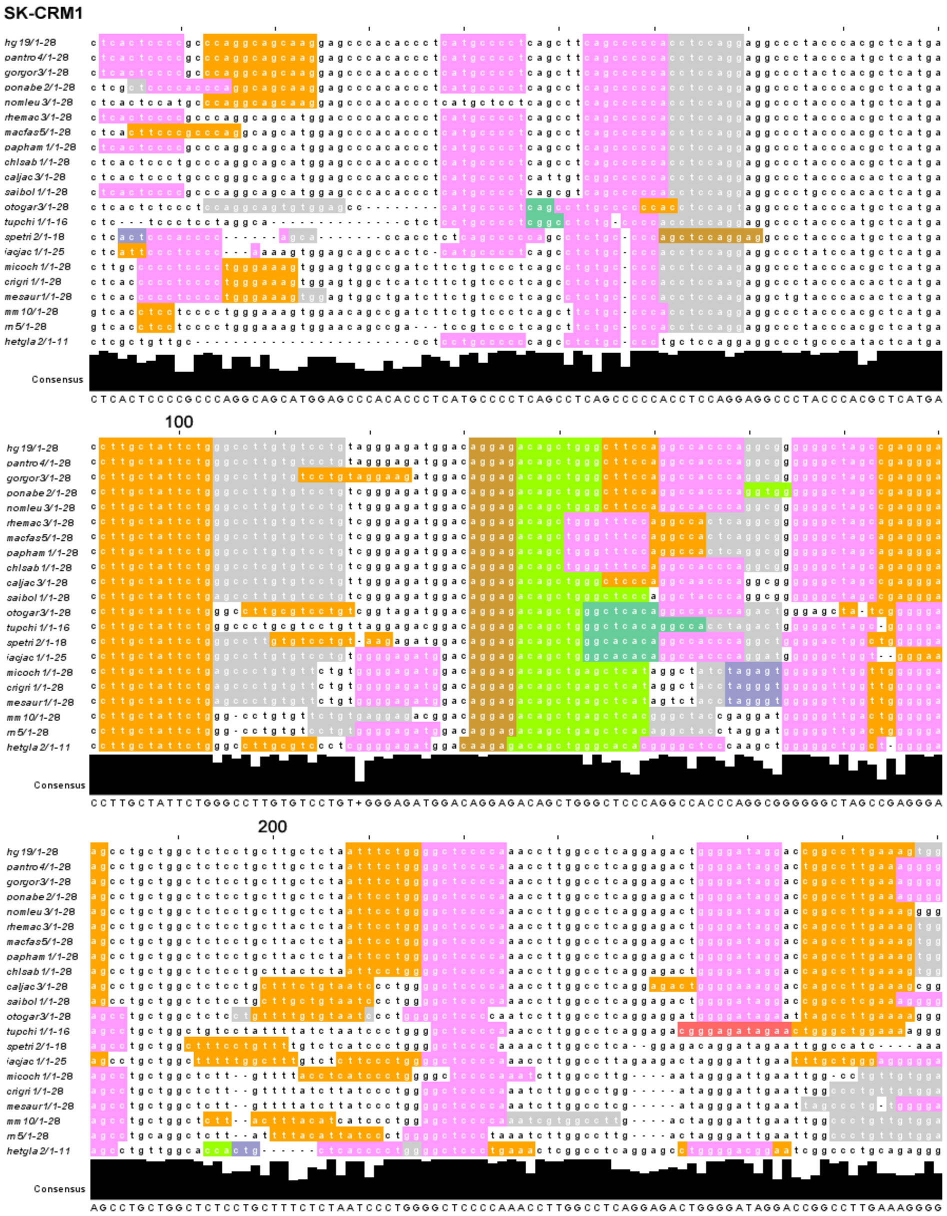

Supplementary Figure 1 (cont.)

a Sk-CRM1 (*ATP2A1* gene) (cont.)

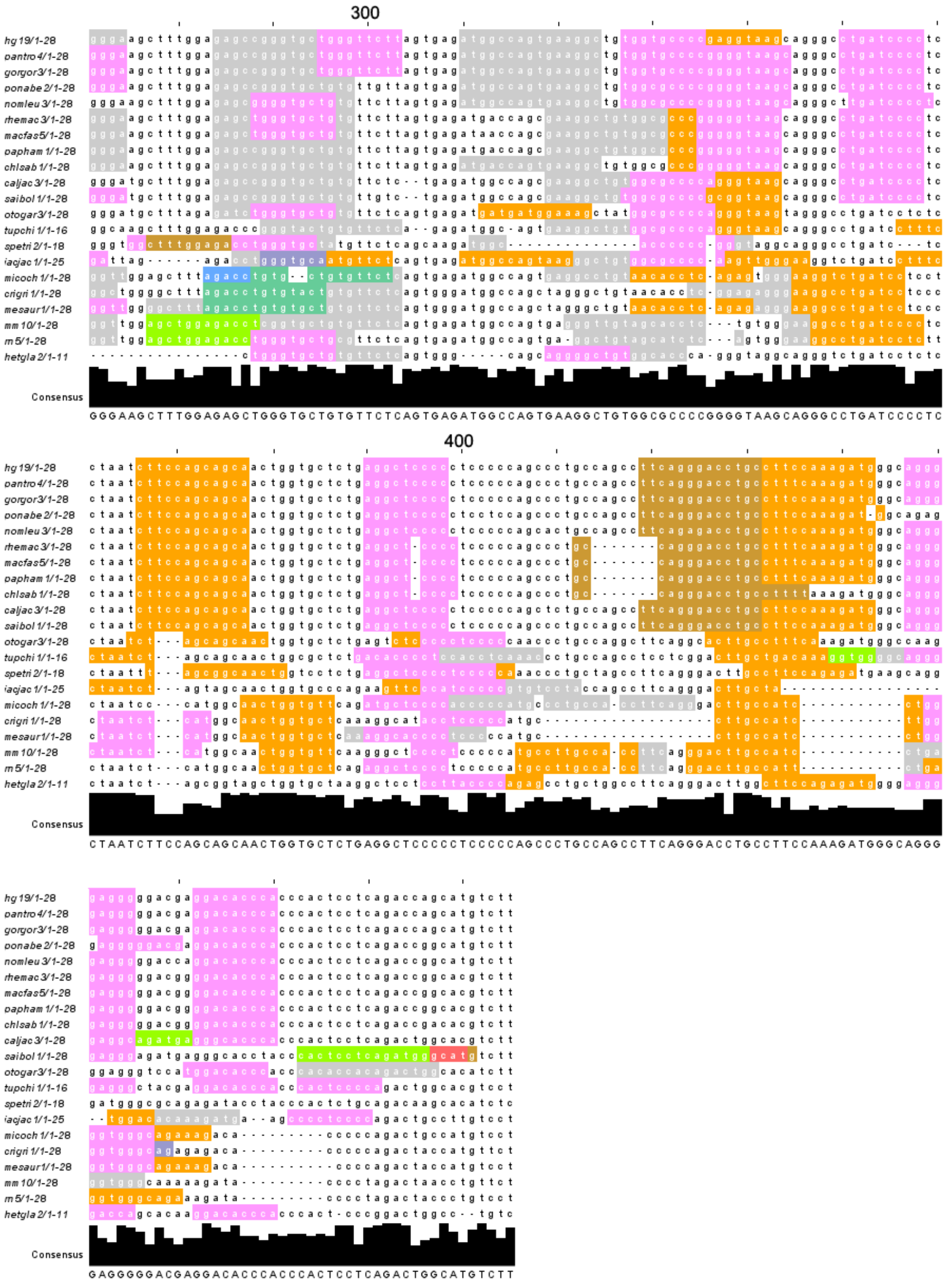

Supplementary Figure 1 (cont.)

b **Sk-CRM2 (*TNNI1*[a] gene)**

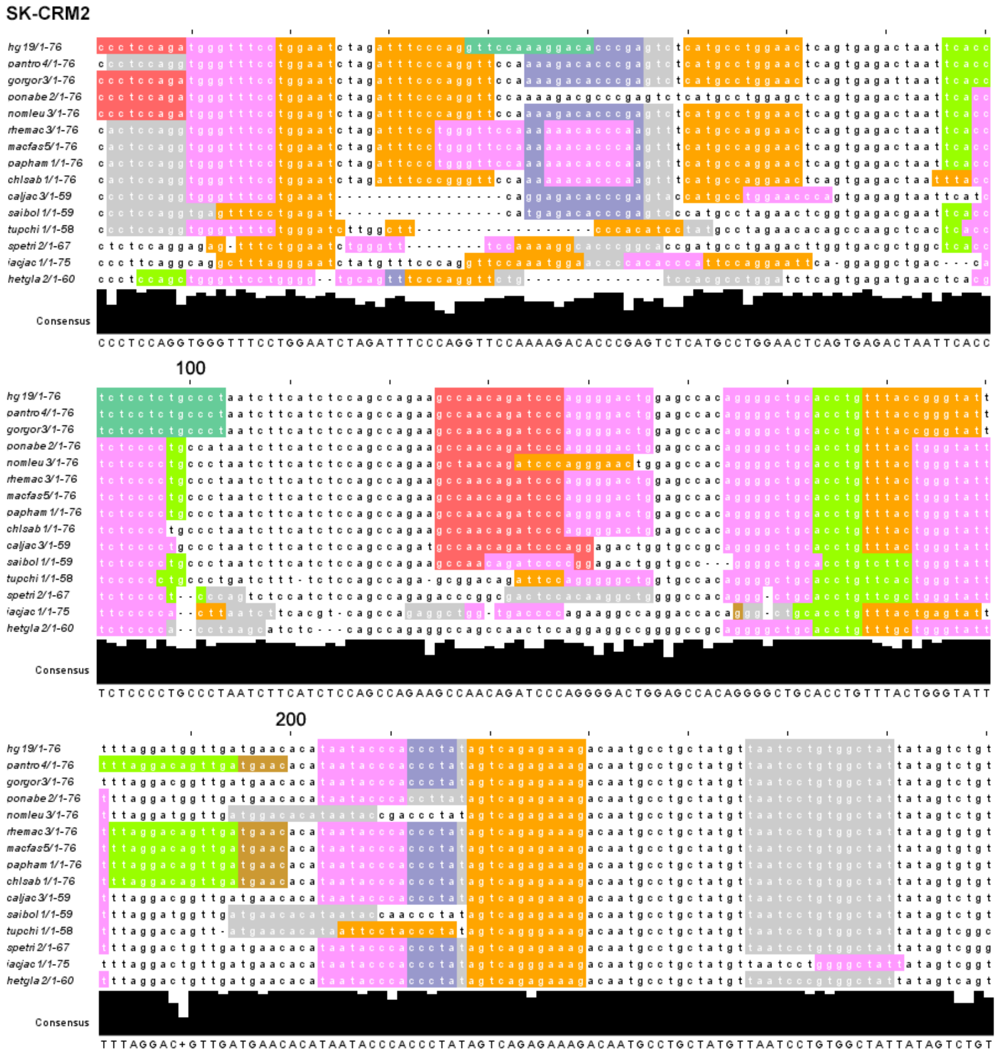

Supplementary Figure 1 (cont.)

c Sk-CRM3 (*TNNI1*[b] gene)

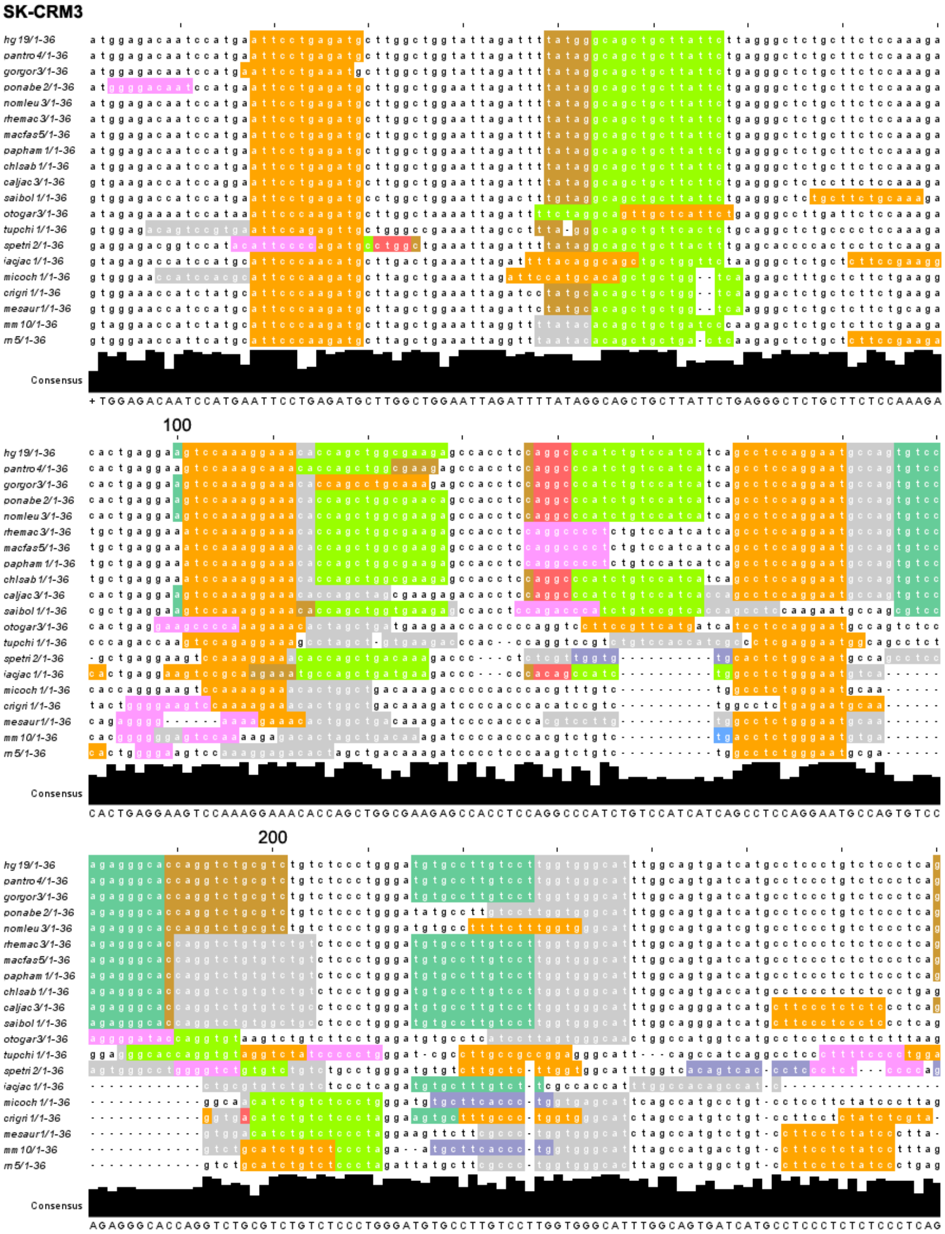

Supplementary Figure 1 (cont.)

c **Sk-CRM3 (*TNNI1*^{b} gene) (cont.)**

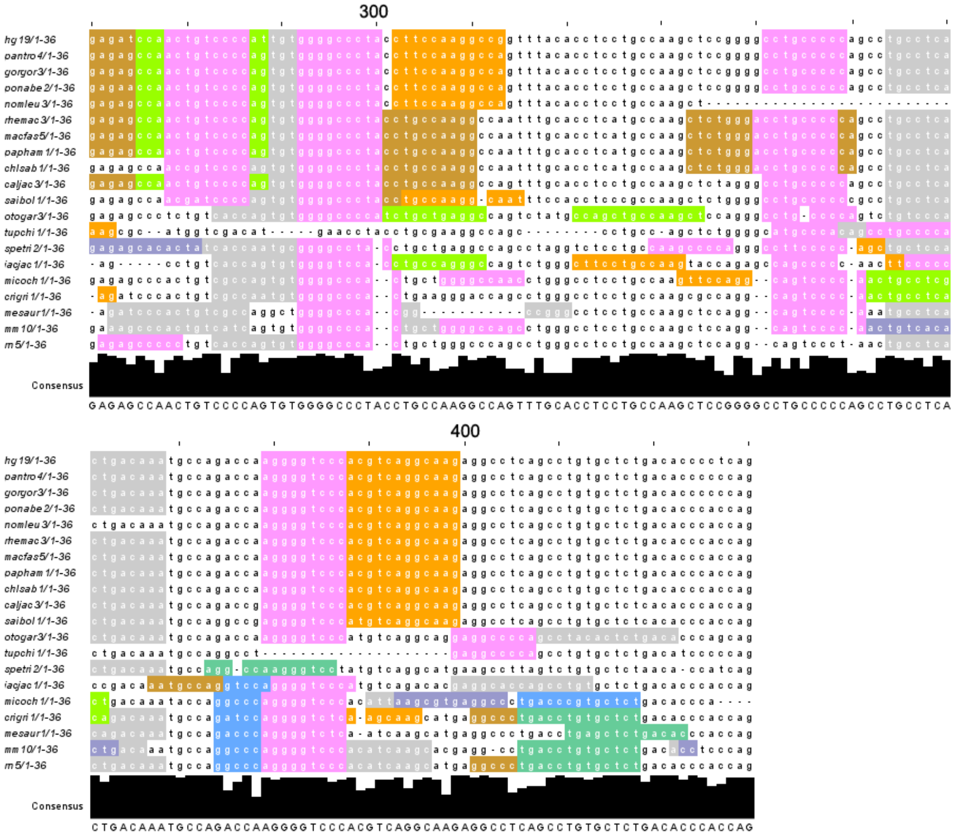

Supplementary Figure 1 (cont.)

d Sk-CRM4 (*MYLPF* gene)

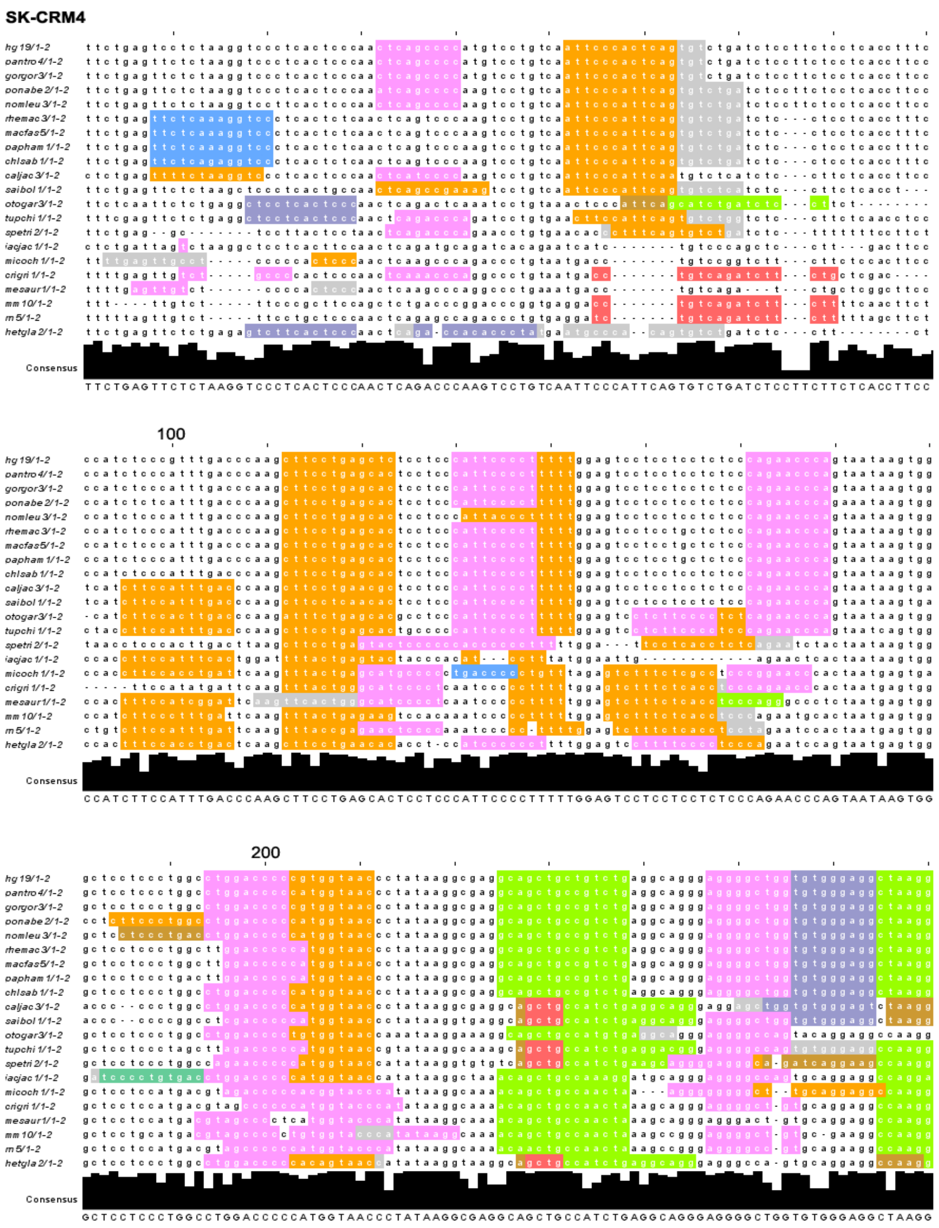

Supplementary Figure 1 (cont.)

e Sk-CRM5 (*MYH1* gene)

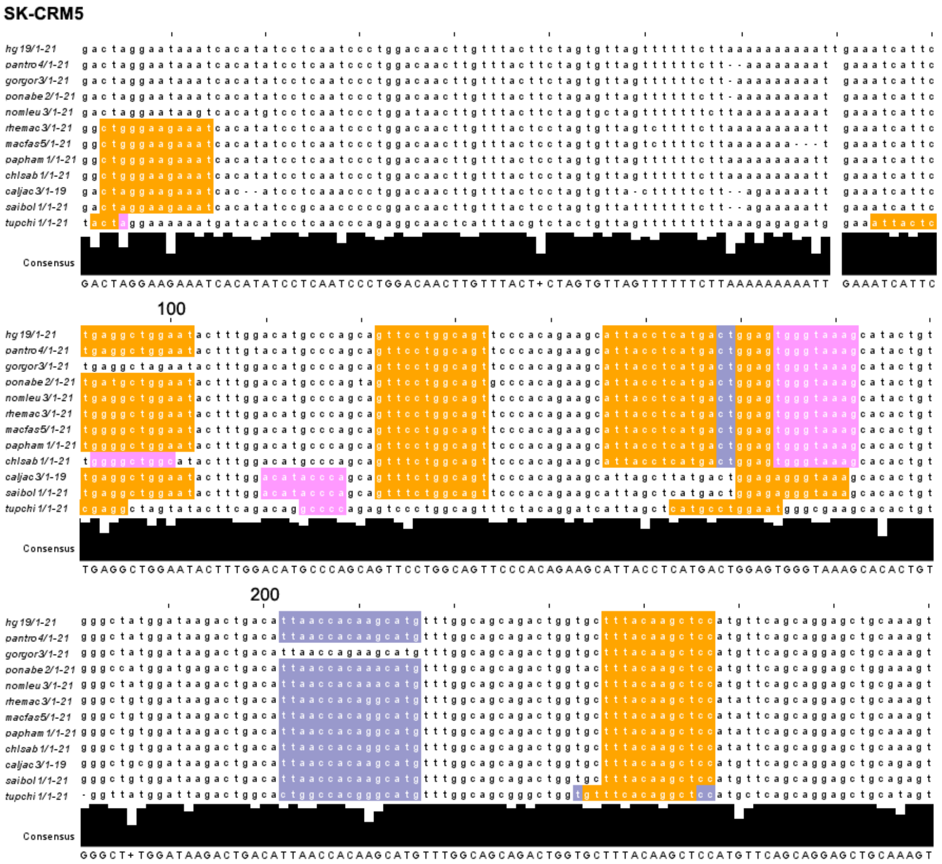

Supplementary Figure 1 (cont.)

e **Sk-CRM5 (*MYH1* gene) (cont.)**

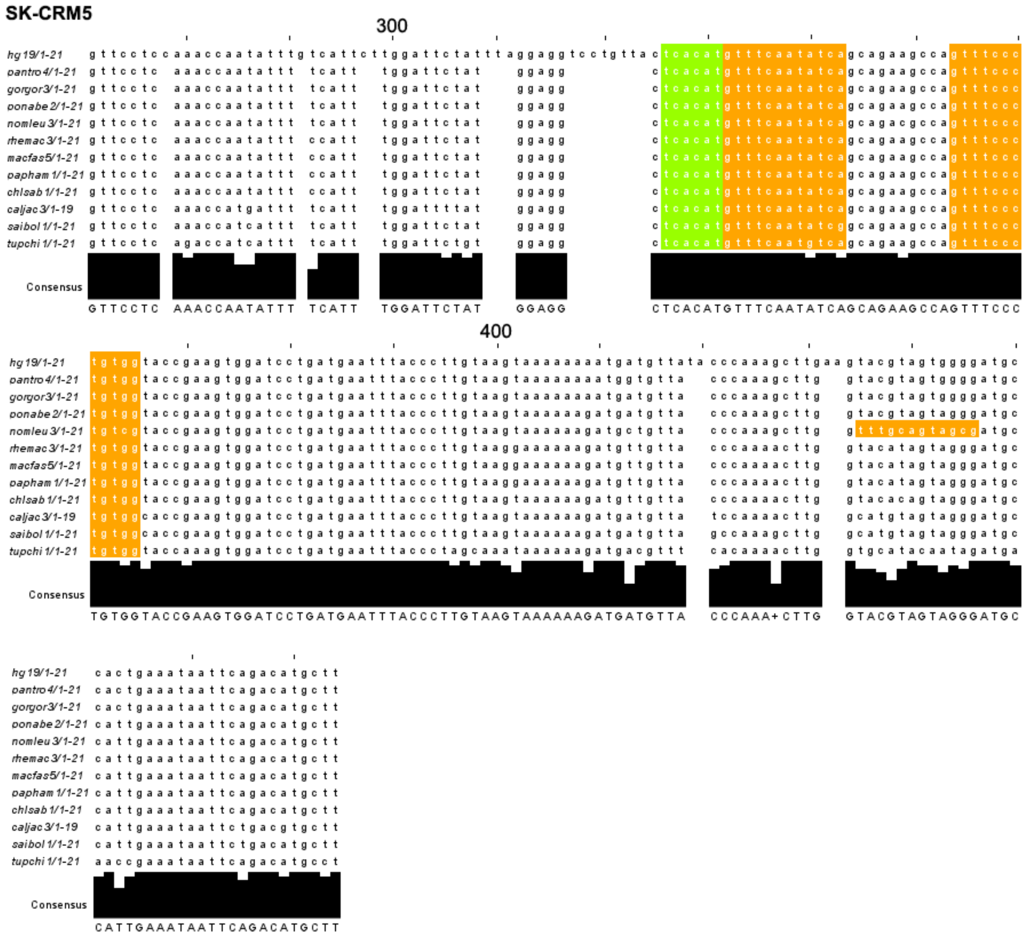

Supplementary Figure 1 (cont.)

f Sk-CRM6 (*TPM3* gene)

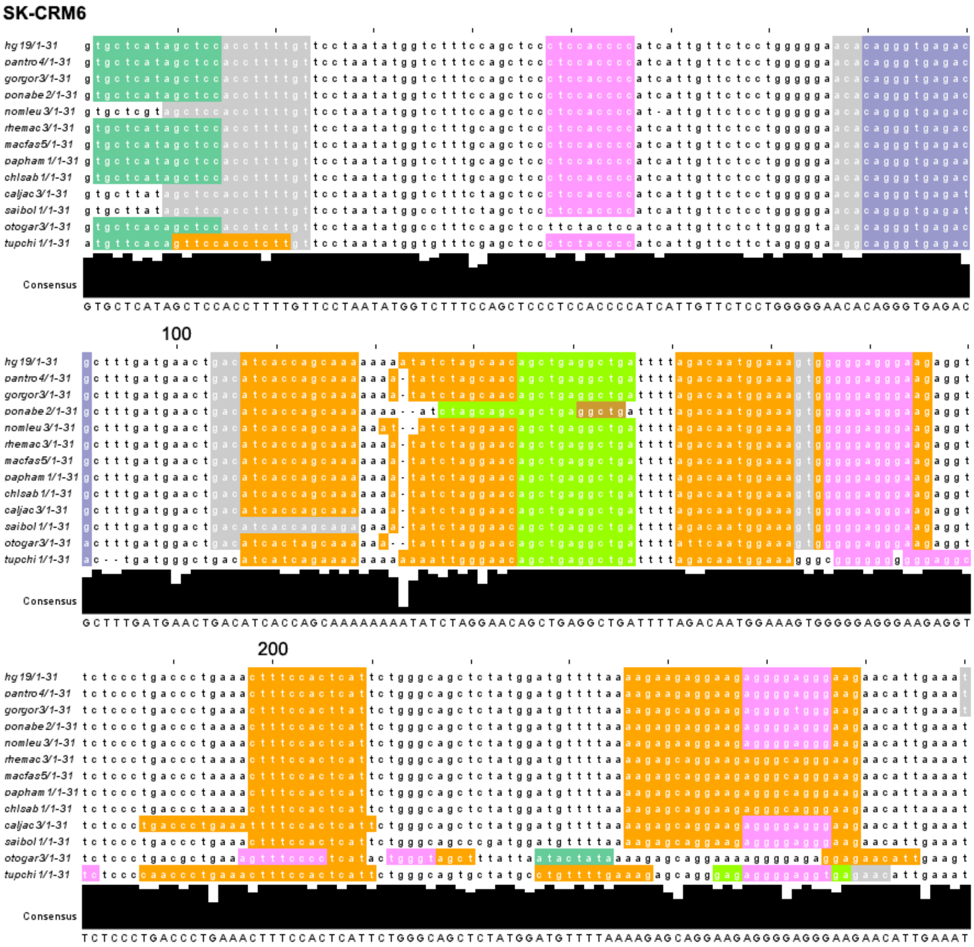

Supplementary Figure 1 (cont.)

f **Sk-CRM6 (*TPM3* gene) (cont.)**

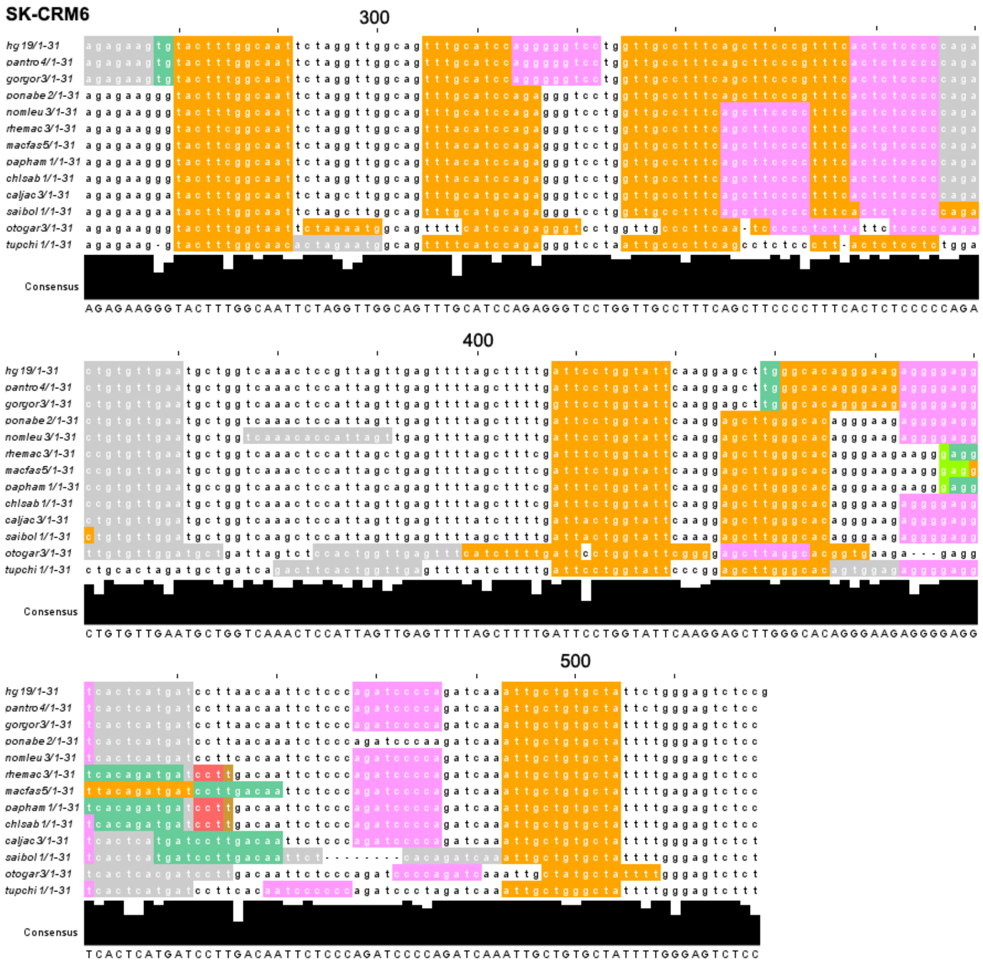

Supplementary Figure 1 (cont.)

g Sk-CRM7 (*ANKRD2* gene)

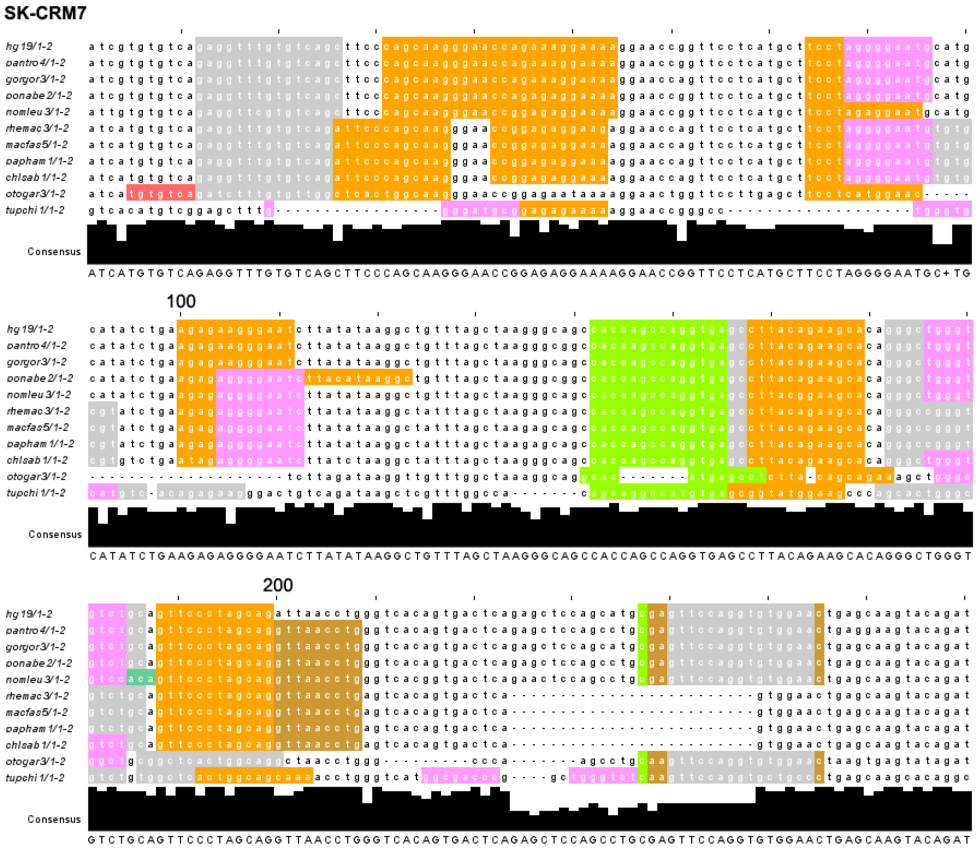

Supplementary Figure 1 (cont.)

g Sk-CRM7 (*ANKRD2* gene) (cont.)

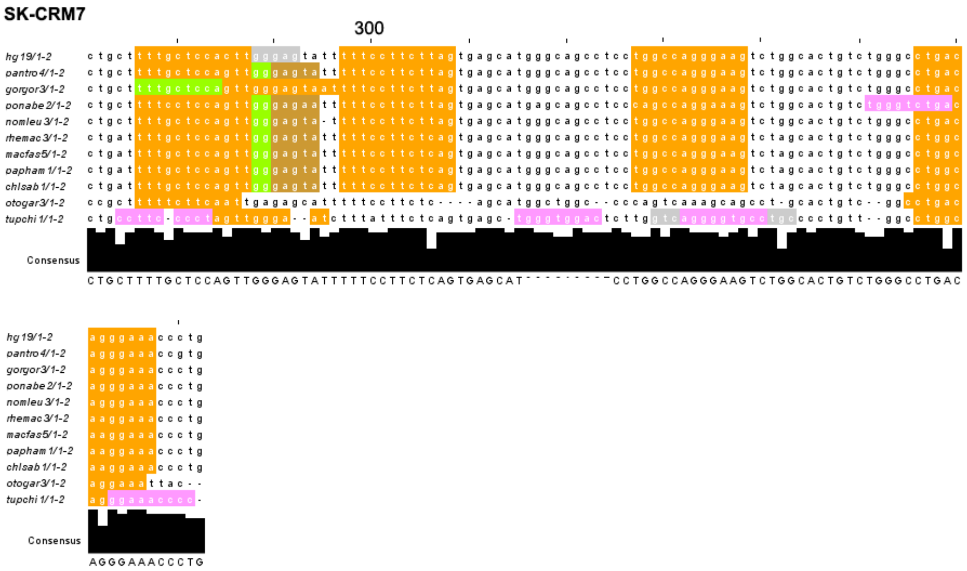

Color code — TF Identifier

- Orange: C/EBP
- Green: E2A
- Light grey / Lavender: SREBP
- Blue: COUP direct repeat 1
- Pink: LRP
- Ochre: MyoD
- Red: Tal-1 beta E47
- Teal: PPAR

Supplementary Figure 1. Schematic representation of the organization of the *Sk-CRM* elements. The evolutionary conservation is highlighted (species shown on the left) and the gene/promoter from which the *Sk-CRM* was derived is indicated. *TFBS* include putative binding sites for E2A, CEBP, LRF, MyoD, SREBP, Tal1, PPAR, as indicated by the different colors.

Supplementary Figure 2

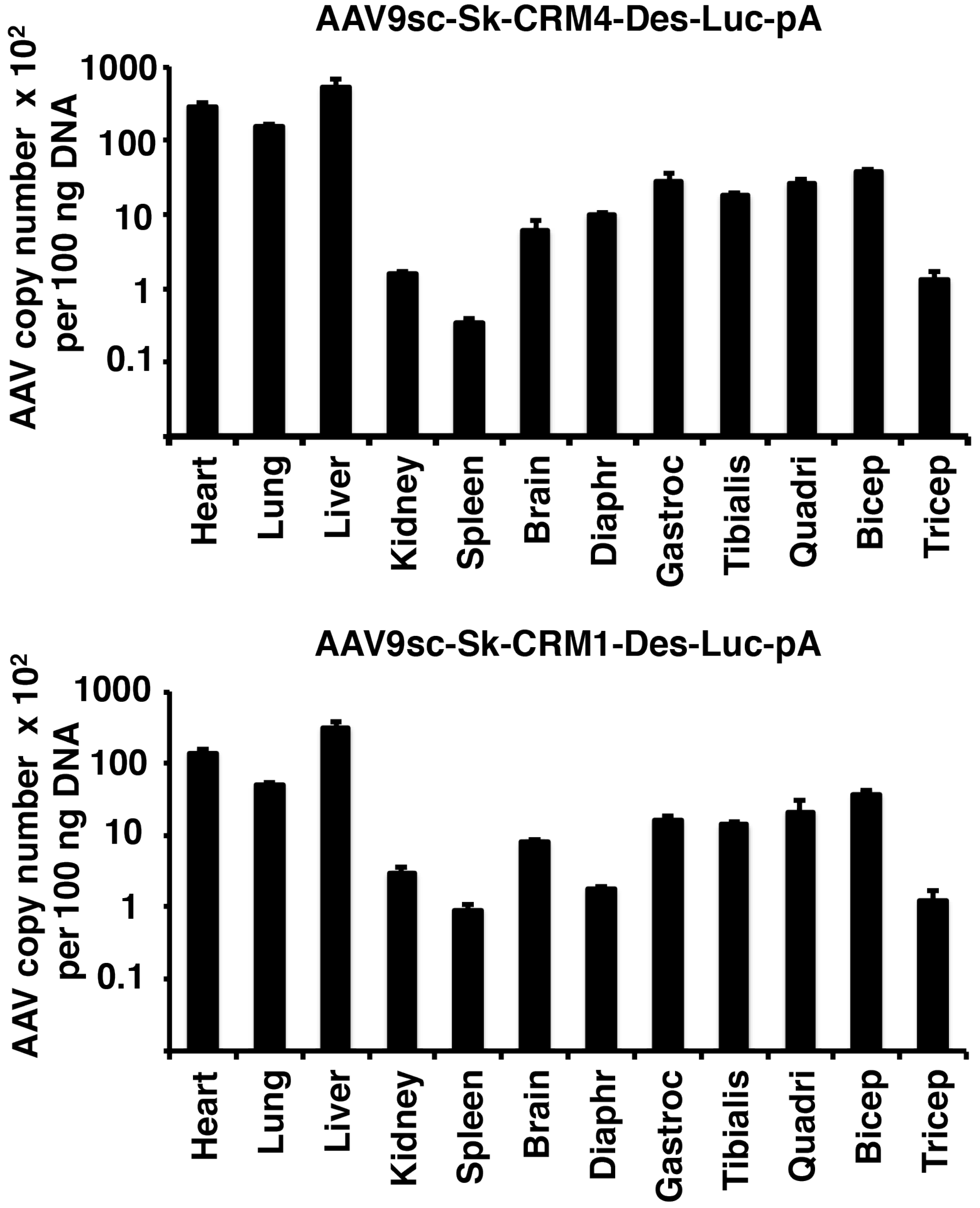

Supplementary Figure 2. Biodistribution and transduction efficiency analysis. AAV copy number was determined by qPCR per 100 ng of DNA in different organs of mice injected with scAAV9-Sk-CRM4-Des-Luc (*Sk-CRM4*) (top panel) and scAAV9-Sk-CRM1-Des-Luc (*Sk-CRM1*) (bottom panel). The data were represented as mean ± s.e.m..

Supplementary Figure 3

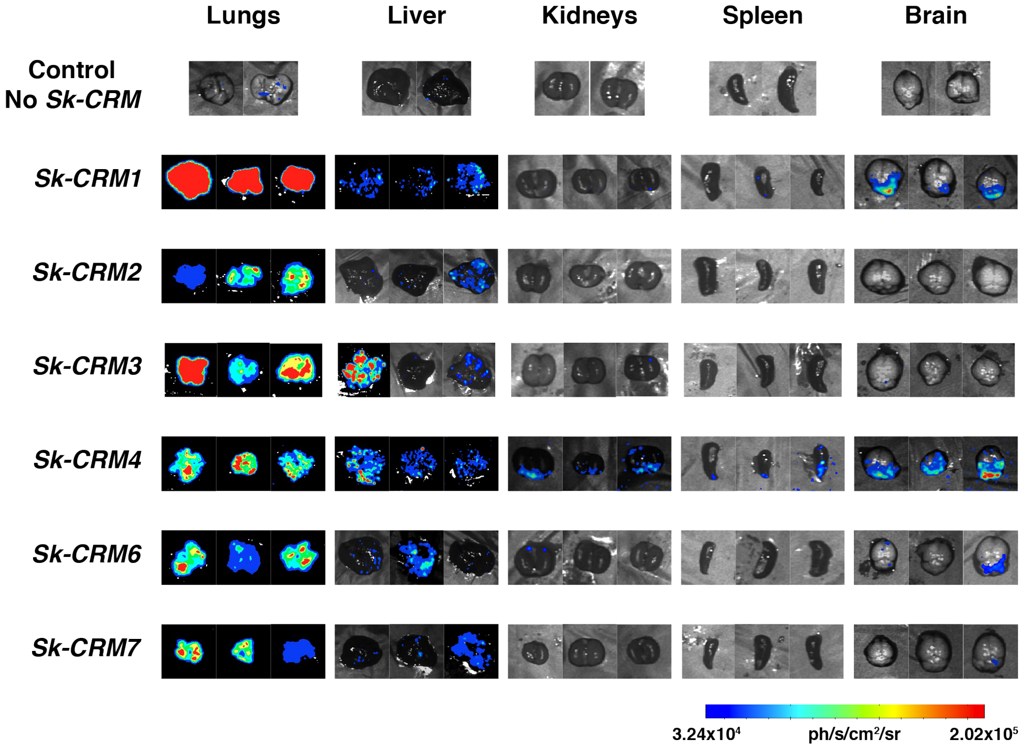

Supplementary Figure 3. Tissue-specificity of *Sk-CRM* determined by *ex vivo* analysis of luciferase expression in lung, liver, kidney, spleen and brain (a) *Ex vivo* bioluminescence imaging from neonatal SCID mice injected intravenously with scAAV9-luciferase vectors (scAAV9-Des-Luc, scAAV9-*Sk-CRM1*-Des-Luc, scAAV9-*Sk-CRM2*-Des-Luc, scAAV9-*Sk-CRM3*-Des-Luc, scAAV9-*Sk-CRM4*-Des-Luc, scAAV9-*Sk-CRM6*-Des-Luc, scAAV9-*Sk-CRM7*-Des-Luc; 5×10^9 vg/mouse) containing the different *Sk-CRMs* or the *Des* promoter without the *Sk-CRM*. Images of the harvested tissues and organs were taken 7 weeks post vector injection. Bioluminescent images of all tissues and organs were represented based on a color scale, showing intensities ranging from 3.23e+04 (blue) ph/s/cm^2/sr to 2.02e+05 (red) ph/s/cm^2/sr.

Supplementary Figure 4

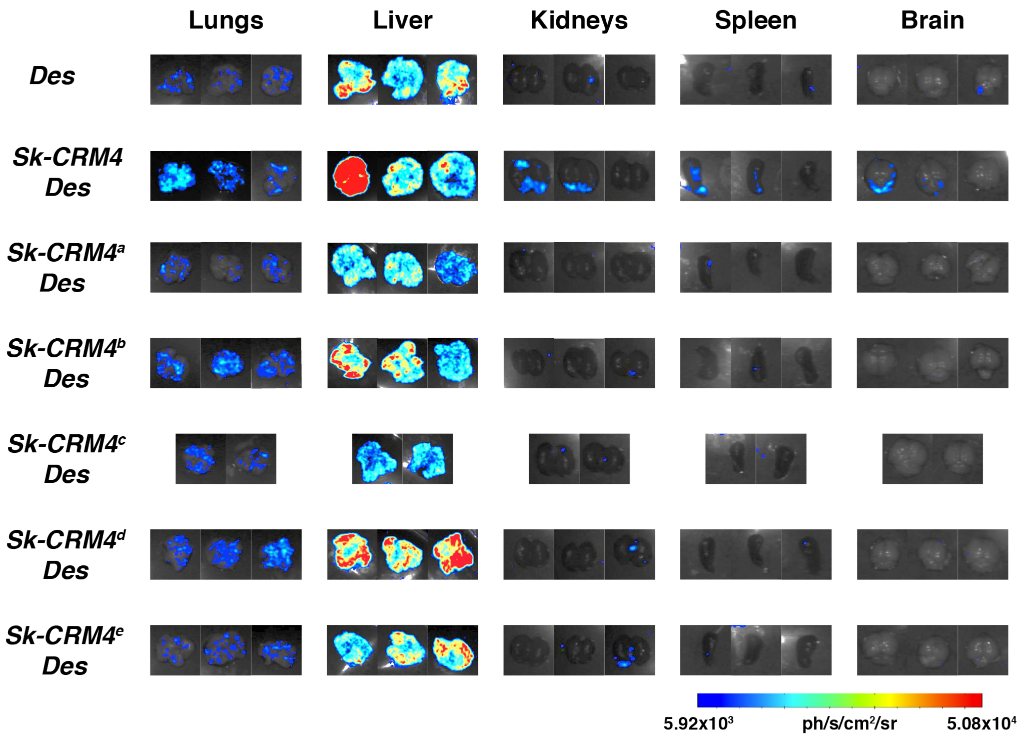

Supplementary Figure 4. Tissue-specificity of *Sk-CRM* fragments. *Ex vivo* bioluminescence imaging of different tissues and organs (lung, liver, kidney, spleen and brain) from SCID mice injected intravenously with scAAV9-luciferase vectors; 10^{10} vg/mouse) containing *Sk-CRM4* (i.e. scAAV9-SkCRM4-Des-Luc) or the different subfragments of *Sk-CRM4* (i.e. scAAV9-SkCRM4^{a}-Des-Luc, scAAV9-SkCRM4^{b}-Des-Luc, scAAV9-SkCRM4^{c}-Des-Luc, scAAV9-SkCRM4^{d}-Des-Luc, scAAV9-SkCRM4^{e}-Des-Luc) or the *Des* promoter as such without *Sk-CRM* (i.e. scAAV9-Des-Luc). Images of all tissues and organs were taken 5 weeks post vector injection. Bioluminescent images of all tissues and organs were represented based on a color scale, showing intensities ranging from $5.92X10^{3}$ (blue) ph/s/cm^{2}/sr – $5.08X10^{4}$ (red) ph/s/cm^{2}/sr.

Supplementary Figure 5

MD1Δ sequence

ATGCTGTGGTGGGAGGAAGTGGAGGACTGCTACGAGAGAGAGGACGTGCAGAAGAAAACCTTCACCAAGTGGG
TGAACGCCCAGTTCAGCAAGTTCGGCAAGCAGCACATCGAGAACCTGTTCAGCGACCTGCAGGATGGCAGGAG
ACTGCTGGATCTGCTGGAGGGACTGACCGGCCAGAAGCTGCCCAAGGAGAAGGGCAGCACCAGAGTGCACGC
CCTGAACAACGTGAACAAGGCCCTGAGAGTGCTGCAGAACAACAACGTGGACCTGGTGAATATCGGCAGCACC
GACATCGTGGACGGCAACCACAAGCTGACCCTGGGCCTGATCTGGAACATCATCCTGCACTGGCAGGTGAAGAA
CGTGATGAAGAACATCATGGCCGGCCTGCAGCAGACCAACAGCGAGAAGATCCTGCTGAGCTGGGTGAGGCAG
AGCACCAGAAACTACCCCCAGGTGAACGTGATCAACTTCACCACCTCCTGGAGCGACGGCCTGGCCCTGAACG
CCCTGATCCACAGCCACAGACCCGACCTGTTCGACTGGAACAGCGTGGTGTGTCAGCAGAGCGCCACCCAGAG
ACTGGAGCACGCCTTCAACATCGCCAGATACCAGCTGGGCATCGAGAAGCTGCTGGACCCCGAGGACGTGGAC
ACCACCTACCCCGACAAGAAAAGCATCCTGATGTATATTACCTCTCTGTTTCAGGTGCTGCCCCAGCAGGTGTCC
ATCGAGGCCATCCAGGAAGTGGAAATGCTGCCCAGGCCCCCCACCGTGTCCCTGGCCCAGGGCTATGAGAGAA
CCAGCAGCCCCAAGCCCAGATTCAAGAGCACCGTGTCCCTGGCCCAGGGCTATGAGAGAACCAGCAGCCCCAA
GCCCAGATTCAAGAGCTACGCCTACACCCAGGCCGCCTACGTGACCACCTCCGACCCCACCAGAAGCCCCTTCC
CCAGCCAGCACCTGGAGGCCCCCGAGGACAAGAGCTTCGGCAGCAGCCTGATGGAGAGCGAAGTGAACCTGG
ACAGATACCAGACCGCCCTGGAGGAAGTGCTGTCTTGGCTGCTGTCCGCCGAGGACACCCTGCAGGCCCAGGG
CGAGATCAGCAACGACGTGGAAGTGGTGAAGGACCAGTTCCACACCCACGAGGGCTACATGATGGATCTGACC
GCCCACCAGGGCAGAGTGGGCAATATCCTGCAGCTGGGCAGCAAGCTGATCGGCACCGGCAAGCTGAGCGAG
GACGAGGAGACCGAAGTGCAGGAGCAGATGAACCTGCTGAACAGCAGATGGGAGTGCCTGAGAGTGGCCAGCA
TGGAGAAGCAGAGCAACCTGCACCGCGTGCTGATGGACCTGCAGAACCAGAAGCTGAAGGAGCTGAACGACTG
GCTGACCAAGACCGAGGAGCGGACCAGAAAGATGGAGGAGGAGCCCCTGGGCCCCTGGGAGAGAGCCATCTC
CCCCAACAAAGTGCCCTACTACATCAACCACGAGACCCAGACCACCTGCTGGGACCACCCTAAGATGACCGAGC
TGTACCAGAGCCTGGCCGACCTGAACAATGTGCGGTTCAGCGCCTACAGAACCGCCATGAAGCTGCGGAGACT
GCAGAAGGCCCTGTGCCTGGACCTGCTGAGCCTGAGCGCCGCCTGCGACGCCCTGGACCAGCACAACCTGAAG
CAGAACGACCAGCCCATGGACATTCTGCAGATCATCAACTGCCTGACCACCATCTACGATCGGCTGGAGCAGGA
GCACAACAACCTGGTGAACGTGCCCCTGTGCGTGGACATGTGCCTGAATTGGCTGCTGAACGTGTACGACACCG
GCAGGACCGGCAGAATCAGAGTGCTGTCCTTCAAGACCGGCATCATCAGCCTGTGCAAGGCCCACCTGGAGGA
TAAGTACCGCTACCTGTTCAAGCAGGTGGCCAGCAGCACCGGCTTCTGCGATCAGAGGAGACTGGGCCTGCTG
CTGCACGATAGCATCCAGATCCCTAGGCAGCTGGGCGAAGTGGCCAGCTTTGGCGGCAGCAACATCGAGCCCT
CTGTGAGGAGCTGCTTCCAGTTCGCCAACAACAAGCCCGAGATCGAGGCCGCCCTGTTCCTGGATTGGATGAGG
CTGGAGCCCCAGAGCATGGTGTGGCTGCCTGTGCTGCACAGAGTGGCCGCCGCCGAGACCGCCAAGCACCAG
GCCAAGTGCAACATCTGCAAGGAGTGCCCCATCATCGGCTTCCGGTACAGGAGCCTGAAGCACTTCAACTACGA
CATCTGCCAGAGCTGCTTTTTCAGCGGCAGAGTGGCCAAGGGCCACAAGATGCACTACCCCATGGTGGAGTACT
GCACCCCCACCACCTCCGGCGAGGATGTGAGAGACTTCGCCAAAGTGCTGAAGAATAAGTTCCGGACCAAGCG
GTACTTTGCCAAGCACCCCAGGATGGGCTACCTGCCCGTGCAGACCGTGCTGGAGGGCGACAACATGGAGACC
GACACCATGTGATGATGA

Supplementary Figure 5. Sequence of truncated micro-dystrophin (*MD1Δ*) containing additional deletions of the spectrin-like repeats

Supplementary Figure 6

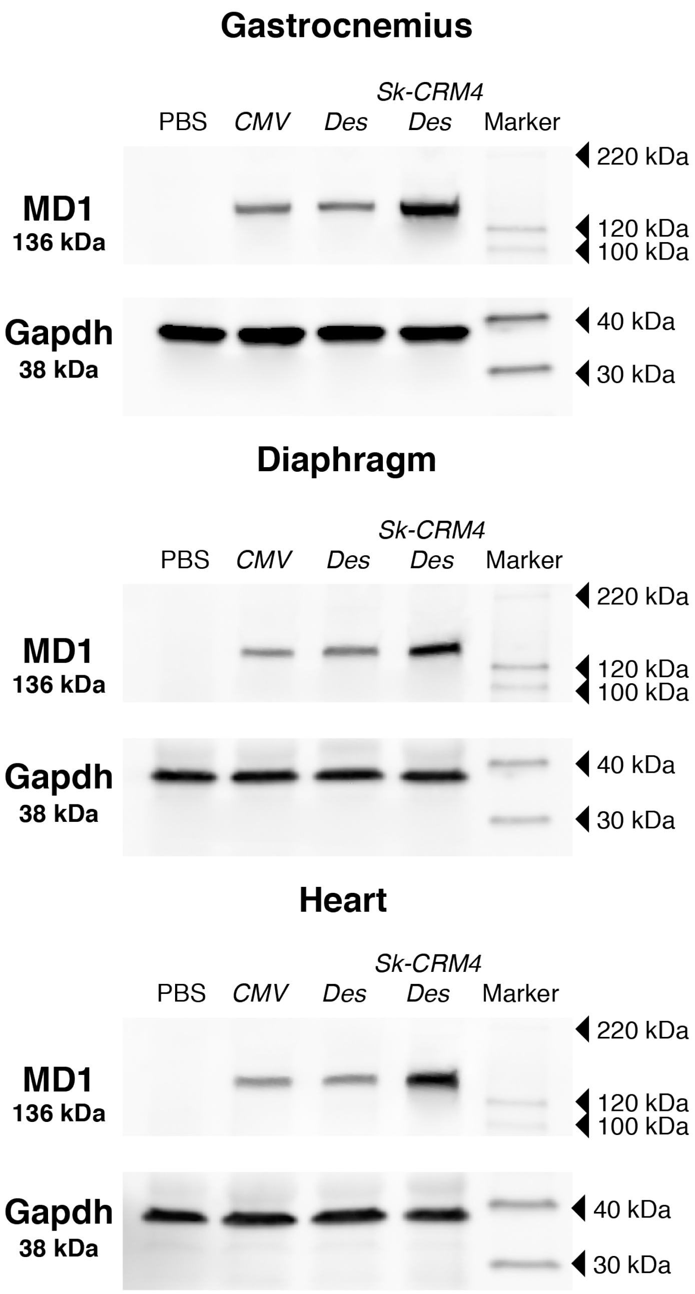

Supplementary Figure 6. Western blot analysis in heart, diaphragm and skeletal muscle. The MD1 proteins in heart, diaphragm and skeletal muscle (*gastrocnemius*) were detected of treated and control SCID/mdx using dystrohin-specific (DYS3) antibody resulting in MD1 (136 kDa) bands. The protein markers were shown to indicate the sizes of MD1 as well as mouse Gapdh (38 kDa) proteins.

Supplementary Figure 7

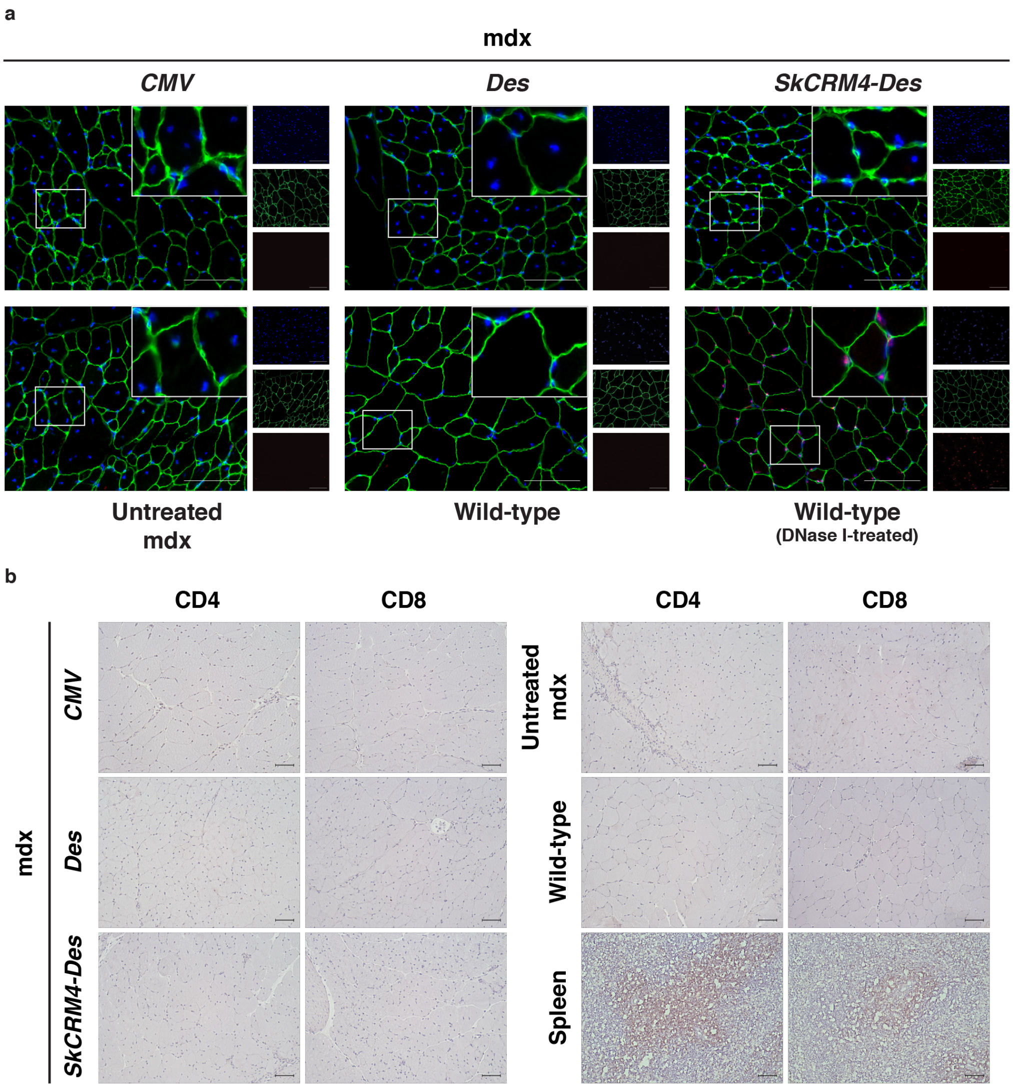

Supplementary Figure 7: Assessments of immune invasion as cellular apoptosis in skeletal muscles after treatments. Mdx mice were injected with *Sk-CRM4/Des* chimeric promoter or conventional promoters at a dose of 5×10^{11} vg/mouse. **(a)** TUNEL assay of 5 μm thick transverse sections of the tibialis anterior muscle at 16-week post-injection of mdx mice. The skeletal muscles from wild-type treated with DNase I were as a positive control. Blue: DAPI staining, Green: Laminin staining, Red: DNA-nicked fragment. The scale bars indicate 100 μm. **(b)** Immunohistochemistry of tibialis anterior muscle from mdx mice after AAV vector injection, as indicated. The CD4 and CD8-positive cells were stained using CD4 and CD8-specifc antibodies and visualized using DAB with hematoxylin counterstaining. Spleens from wild-type mice were used as positive control for CD4 and CD8 detection. The scale bars indicate 20 μm.

Supplementary Table 1

Name	Gene	Length (bp)	TFBS	Sequence	Position in hg19 genome	Distance related to the gene
**Sk-CRM1**	*ATP2A1*	495	E2A, CEBP, LRF, MyoD	CTCACTCCCCGCCCAGGCAGCAAGGAGCCCACACCCTCATGCCCC<br>TCAGCTTCAGCCCCCACCTCCAGGAGGCCCTACCCACGCTCATGAC<br>CTTGCTATTCTGGGCCTTGTGTCCTGTAGGGAGATGGACAGGAGAC<br>AGCTGGGCTTCCAGGCCACCCAGGCGGGGGGCTAGCCGAGGGAA<br>GCCTGCTGGCTCTCCTGCTTGCTCTAATTTCTGGGGCTCCCCAAAC<br>CTTGGCCTCAGGAGACTGGGGATAGGACCGGCCTTGAAAGTGGGG<br>GAAGCTTTGGAGAGCCGGGTGCTGGGTTCTTAGTGAGATGGCCAGT<br>GAAGGCTGTGGTGCCCCGAGGTAAGCAGGGCCTGATCCCCTCCTA<br>ATCTTCCAGCAGCAACTGGTGCTCTGAGGCTCCCCCTCCCCCAGCC<br>CTGCCAGCCTTCAGGGACCTGCCTTCCAAAGATGGGCAGGGGAGG<br>GGGACGAGGACACCCACCCACTCCTCAGACCAGCATGTCTT	chr16:28,886,777-28,887,271	2,538 bp to *ATP2A1* TSS
**Sk-CRM2**	*TNNI1*[a]	344	E2A, CEBP, LRF, MyoD, SREBP	CCCTCCAGATGGGTTTCCTGGAATCTAGATTTCCCAGGTTCCAAAG<br>GACACCCGAGTCTCATGCCTGGAACTCAGTGAGACTAATTCACCTC<br>TCCTCTGCCCTAATCTTCATCTCCAGCCAGAAGCCAACAGATCCCAG<br>GGGACTGGAGCCACAGGGGCTGCACCTGTTTACCGGGTATTTTTAG<br>GATGGTTGATGAACACATAATACCCACCCTATAGTCAGAGAAAGACA<br>ATGCCTGCTATGTTAATCCTGTGGCTATTATAGTCTGTCATCTCATG<br>GGTTGGGGCAGGACACTGACCCTCTCAGAGGCCAGAGAGAGGCCT<br>CGCAAGCAGGAGGTTAGGGA	chr1:201,391,575-201,391,918	701 bp to *TNNI1* TSS
**Sk-CRM3**	*TNNI1*[b]	430	E2A, CEBP, LRF, MyoD, SREBP, Tal1	ATGGAGACAATCCATGAATTCCTGAGATGCTTGGCTGGTATTAGATT<br>TTATGGGCAGCTGCTTATTCTTAGGGCTCTGCTTCTCCAAAGACACT<br>GAGGAAGTCCAAAGGAACACCAGCTGGCGAAGAGCCACCTCCAGG<br>CCCATCTGTCCATCATCAGCCTCCAGGAATGCCAGTGTCCAGAGGG<br>CACCAGGTCTGCGTCTGTCTCCCTGGGATGTGCCTTGTCCTTGGTG<br>GGCATTTGGCAGTGATCATGCCTCCCTGTCTCCCTCAGAGATCCAA<br>CTGTCCCCATTGTGGGGCCCTACCTTCCAAGGCCGGTTTACACCTC<br>CTGCCAAGCTCCGGGGCCTGCCCCCAGCCTGCCTCACTGACAAAT<br>GCCAGACCAAGGGGTCCCACGTCAGGCAAGAGGCCTCAGCCTGTG<br>CTCTGACACCCCTCAG	chr1:201,388,987-201,389,416	1,438 bp to *TNNI1* TSS

Name	Gene	Length (bp)	TFBS	Sequence	Position in hg19 genome	Distance related to the gene
**Sk-CRM4**	*MYLPF*	435	E2A, CEBP, LRF, MyoD, SREBP	TTCTGAGTCCTCTAAGGTCCCTCACTCCCAACTCAGCCCCATGTCCT<br>GTCAATTCCCACTCAGTGTCTGATCTCCTTCTCCTCACCTTTCCCAT<br>CTCCCGTTTGACCCAGCTTCCTGAGCTCTCCTCCCATTCCCCTTTTT<br>GGAGTCCTCCTCCTCTCCCAGAACCCAGTAATAAGTGGGCTCCTCC<br>CTGGCCTGGACCCCCGTGGTAACCCTATAAGGCGAGGCAGCTGCT<br>GTCTGAGGCAGGGAGGGGCTGGTGTGGGAGGCTAAGGGCAGCTG<br>CTAAGTTTAGGGTGGCTCCTTCTCTCTTCTTAGAGACAACAGGTGGC<br>TGGGGCCTCAGTGCCCAGAAAAGAAAATGTCTTAGAGGTATCGGCA<br>TGGGCCTGGAGGAGGGGGGACAGGGCAGGGGGAGGCATCTTCCT<br>CAGGACATCGGGTCCTAGAGG	chr16:30,383,321-30,383,755	2,368 bp to *MYLPF* TSS
**Sk-CRM4[a]**	*MYLPF*	171	CEBP, E2A, LRF	CTCTAAGGTCCCTCACTCCCAACTCAGCCCCATGTCCTGTCAATTCC<br>CACTCAGTGTCTGATCTCCTTCTCCTCACCTTTCCCATCTCCCGTTT<br>GACCCAAGCTTCCTGAGCTCTCCTCCCATTCCCCTTTTTGGAGTCCT<br>CCTCCTCTCCCAGAACCCAGTAATAAGTGG	chr16:30,383,330-30,383,500	2,623 bp to *MYLPF* TSS
**Sk-CRM4[b]**	*MYLPF*	51	CEBP, E2A, LRF	TGGCCTGGACCCCCGTGGTAACCCTATAAGGCGAGGCAGCTGCTG<br>TCTGAG	chr16:30,383,510-30,383,560	2,563 bp to *MYLPF* TSS
**Sk-CRM4[c]**	*MYLPF*	60	E2A, LRF, MyoD	GCAGGGAGGGGCTGGTGTGGGAGGCTAAGGGCAGCTGCTAAGTTT<br>AGGGTGGCTCCTTCT	chr16:30,383,561-30,383,620	2,503 bp to *MYLPF* TSS
**Sk-CRM4[d]**	*MYLPF*	41	LRF, MyoD	AGGAGGGGGGACAGGGCAGGGGGAGGCATCTTCCTCAGGAC	chr16:30,383,700-30,383,740	2,383 bp to *MYLPF* TSS
**Sk-CRM4[e]**	*MYLPF*	120	CEBP, E2A, LRF	GCAGGGAGGGGCTGGTGTGGGAGGCTAAGGGCAGCTGCTAAGTTT<br>AGGGTGGCTCCTTCTCTCTTCTTAGAGACAACAGGTGGCTGGGGCC<br>TCAGTGCCCAGAAAAGAAAATGTCTTAGA	chr16:30,383,561-30,383,680	2,443 bp to *MYLPF* TSS

Name	Gene	Length (bp)	TFBS	Sequence	Position in hg19 genome	Distance related to the gene
**Sk-CRM5**	*MYH1*	474	PPAR, CEBP, LRF, SREBP	GACTAGGAATAAATCACATATCCTCAATCCCTGGACAACTTGTTTAC<br>TTCTAGTGTTAGTTTTTTCTTAAAAAAAAAATTGAAATCATTCTGAGG<br>CTGGAATACTTTGGACATGCCCAGCAGTTCCTGGCAGTTCCCACAG<br>AAGCATTACCTCATGACTGGAGTGGGTAAAGCATACTGTGGGCTAT<br>GGATAAGACTGACATTAACCACAAGCATGTTTGGCAGCAGACTGGT<br>GCTTTACAAGCTCCATGTTCAGCAGGAGCTGCAAAGTGTTCCTCCAA<br>ACCAATATTTGTCATTCTTGGATTCTATTTAGGAGGTCCTGTTACTCA<br>CATGTTTCAATATCAGCAGAAGCCAGTTTCCCTGTGGTACCGAAGTG<br>GATCCTGATGAATTTACCCTTGTAAGTAAAAAAAATGATGTTATACCC<br>AAAGCTTGAAGTACGTAGTGGGGATGCCACTGAAATAATTCAGACAT<br>GCTT	chr17:10,416,614-10,417,087	4,772 bp to *MYH1* TSS
**Sk-CRM6**	*TPM3*	519	E2A, CEBP, LRF, MyoD, SREBP	GTGCTCATAGCTCCACCTTTTGTTCCTAATATGGTCTTTCCAGCTCC<br>CTCCACCCCATCATTGTTCTCCTGGGGGAACACAGGGTGAGACGCT<br>TTGATGAACTGACATCACCAGCAAAAAAAATATCTAGCAACAGCTGA<br>GGCTGATTTTAGACAATGGAAAGTGGGGGAGGGAAGAGGTTCTCCC<br>TGACCCTGAAACTTTCCACTCATTCTGGGCAGCTCTATGGATGTTTT<br>AAAAGAAGAGGAAGAGGGGAGGGAAGAACATTGAAATAGAGAAGT<br>GTACTTTGGCAATTCTAGGTTGGCAGTTTGCATCCAGGGGGTCCTG<br>GTTGCCTTTCAGCTTCCCGTTTCACTCTCCCCCAGACTGTGTTGAAT<br>GCTGGTCAAACTCCGTTAGTTGAGTTTTAGCTTTTGATTCCTGGTAT<br>TCAAGGAGCTTGGGCACAGGGAAGAGGGGAGGTCACTCATGATCC<br>TTAACAATTCTCCCAGATCCCCAGATCAAATTGCTGTGCTATTCTGG<br>GAGTCTCCG	chr1:154,164,610-154,165,128	517 bp to *TPM3* TSS
**Sk-CRM7**	*ANKRD 2*	372	E2A, CEBP, MyoD	ATCGTGTGTCAGAGGTTTGTGTCAGCTTCCCAGCAAGGGAACCAGA<br>AAGGAAAAGGAACCGGTTCCTCATGCTTCCTAGGGGAATGCATGCA<br>TATCTGAAGAGAAGGGAATCTTATATAAGGCTGTTTAGCTAAGGGCA<br>GCCACCAGCCAGGTGAGCCTTACAGAAGCACAGGGCTGGGTGTCT<br>GCAGTTCCCTAGCAGATTAACCTGGGTCACAGTGACTCAGAGCTCC<br>AGCATGCGAGTTCCAGGTGTGGAACTGAGCAAGTACAGATCTGCTT<br>TTGCTCCACTTGGGAGTATTTTTCCTTCTTAGTGAGCATGGGCAGCC<br>TCCTGGCCAGGGAAGTCTGGCACTGTCTGGGCCTGACAGGGAAAC<br>CCTG	chr10:99,331,378-99,331,749	507 bp to *ANKRD2* TSS

TSS: Transcription start site

Supplementary Table 2

matrix ID	x	y	distance to origin	slope	p-value	trend	trend p-value	TF
M00776	-0.61495684	-0.43085342	0.750870551152782	0.700623835649995	0.001	35.0526321722801	0.198	SREBP
M00804	-0.26318989	-0.65422925	0.705184252343864	2.48576892524253	0.011	30.6956771277907	0.276	E2A
M00065	-0.36876793	-0.17368896	0.407624387178155	0.470998006795222	0.013	55.4177527411296	0.041	Tal-1beta:E47
M00762	-0.31487351	-0.2956267	0.431903314472823	0.93887447057709	0.017	58.904199891356	0.076	PPAR,
M00929	-0.023844732	-0.88602736	0.88634815615125	37.1582016522559	0.017	60.4776107602034	0.231	MyoD
M01168	-0.42305829	-0.89893621	0.993511261328672	2.12485189688636	0.021	106.181731817816	0.104	SREBP
M00770	-0.096630944	0.24264045	0.261174132169581	-2.5110015483239	0.029	15.2676070291572	0.258	C/EBP
M01100	-0.063193563	-1.265489	1.26706583709199	20.0255997592666	0.033	174.518856572171	0.066	LRF
M00765	-0.29507474	-0.1488961	0.330513465355464	0.504604697779281	0.045	28.0601713051276	0.212	COUP

SUPPLEMENTARY METHODS

Identification of *Sk-CRM* using genome-wide computational analysis

In general, the initial step in gene expression is the transcription of the gene from DNA into RNA by RNA polymerase at transcription start sites. However, transcription is often weak in the absence of the regulatory DNA regions called *cis*-regulatory modules (CRMs). *CRMs* are operationally defined as DNA sequences containing transcription factor binding sites (*TFBS*), which are clustered into modular structures [1]. Their sequences generally contain short DNA motifs typically a few hundreds base pairs in length that act as binding sites for sequence-specific transcription factors (TF). These proteins recruit co-activators (or co-repressors) such that combined regulatory cues of all bound elements define the activity of *CRMs* and regulate transcription in terms of efficacy and specificity [2,3]. Despite of their crucial role in regulating gene expression, in general *CRMs* remain poorly annotated in sequence genomes and the vast majority of *CRMs* are yet to be characterized. Previously, several studies predicted and identified the *CRMs* which as specific for tissue including muscle, liver, heart and brain [4,5, 6-8, 9] using different approaches. However, none of these studies implicate *CRMs* in the context of gene therapy. The current strategy requires genome-wide computational approaches to identify robust transcriptional *CRMs* associated with high levels of muscle-specific gene expression. Hence, the main advantage of this computational strategy is that it allows for the identification of robust muscle-specific *CRMs* that contain a 'molecular signature' characteristic for achieving high *in vivo* expression in the muscle.

Highly expressed skeletal muscle-specific genes (Fig. 1a), were identified by using the TiGER database (Tissue-specific Gene Expression and Regulation) that is based on human genomic data [10] (http://bioinfo.wilmer.jhu.edu/tiger/; doi:10.1186/1471-2105-9-271). For that purpose, The 325 muscle-specific genes extracted from the TiGER database are sorted based on their expression levels from high to low. There is a relative enrichment of binding sites for transcription factors (TFs) associated with high tissue-specific gene expression in promoters of genes at the top compared to those at the bottom of this list. Increasing the number of

genes/promoters increases the noise to signal ratio and is therefore not desirable. To test this, a bio-computational analysis was conducted by varying the number of genes/promoters extracted from the TiGER database, that fall within the category of high muscle-specific gene expression. In particular, by using a list of the top-ranked 100 genes/promoters, only one TF was identified associated with high muscle-specific gene expression (i.e. *Tal-1beta:E47*) with a p-value < 0.05. In contrast, by restricting the analysis to the top-ranked 29 genes/promoters, 9 TFs were identified associated with high muscle-specific gene expression. Hence, there was a trade-off between signals versus noise as a function of the number of genes/promoters on which the bio-computational analysis was based. We therefore choose to proceed with the analysis based on the top-ranked 29 genes/promoters.

A set of most highly expressed (i.e. 'over-expressed') genes in the skeletal muscle compared to any of the other tissues was identified using this database. Pairwise comparison was done by a two-tailed t-test. Conversely, a set of 'under-expressed' genes was identified, corresponding to those genes that exhibited the lowest expression in these respective organs compared to any of the other tissues. This analysis resulted in a set of 29 over-expressed genes and another set of 29 under-expressed muscle-specific genes.

Next, the RefSeq IDs lists of these 'over-expressed' and 'under-expressed' skeletal muscle-specific genes were used to extract the corresponding promoter sequences upstream the reported transcription start sites (*TSS*) by up to 1 kb (NCBI36/hg18 genome assembly), using the transcription start location data stored in the refGene table of the UCSC Genome Browser (http://genome.ucsc.edu; doi:10.1101/gr.229102) database. Consequently, two sets of skeletal muscle-specific promoter sequences corresponding to promoters of 'over-expressed' or 'under-expressed' genes were identified. In the next step, the promoter sequences were filtered using 'uclust' (http://www.drive5.com/usearch/; doi:10.1093/bioinformatics/btq461) resulting in a non-redundant set of representative promoter sequences. Evidence from computational analysis and *in vitro* experiments support the rationale for choosing promoters up to 1 kb upstream of the *TSS*. The basal promoter and nearby upstream regulatory

elements are typically found within a 1 kb region upstream of the *TSS*. Indeed, the preferred locations for most of the known *TFBS* in the TRANSFAC® (TRANScription FACtor) database (http://www.biobase-international.com/product/transcription -factor-binding-sites) happen to be between -300 and +50 bp relative to the TSS [11]. Additionally, the fact that a vast majority (>90%) of DNA fragments containing regions -550 to +50 relative to the TSS were transcriptionally active (according to the TRANSFAC data sets) was also based on results from luciferase-based transfection assays in four human cultured cell types [12, 4].

The *TFBS* were then mapped to these promoters using the TRANSFAC® database. TRANSFAC® is a manually curated database of eukaryotic transcription factors, their genomic binding sites and DNA binding profiles which is used to predict potential transcription factor binding sites. Positional weight matrices that are derived based on the broad compilation of binding sites, can be used with either the MatchTM of FIMO tool to search DNA sequences for predicted transcription factor binding sites [13]. Subsequently, the DDM/MDS method, described in detail elsewhere [14], was used on the *TFBS* datasets obtained from the 'over-expressed' versus 'under-expressed' skeletal muscle-specific genes. The computer source code was deposited in a public repository http://www.dmbr.ugent.be/prx/bioit2-public/TFdiff/TFdiff.tar.gz. (doi: 10.1186/gb-2007-8-5-r83) The identification of *TFBS* that are over-represented as well as those that tend to cluster together ('co-occurrence') in promoters of the highly expressed skeletal muscle-specific genes was possible by this method.

In this study, the conserved elements are identified from 100 vertebrate species. For this purpose, the sequences of all conserved sequence elements in the NCBI36/hg18 genome assembly were downloaded based on the information stored in the phastConsElements44way table of the UCSC Genome Browser (http://genome.ucsc.edu) database. The multiple alignments at the basis of this table were generated using 'multiz' [15]. Thus, the phastConsElements44way table contains information about conserved elements identified by phastCons [16]. PhastCons is a hidden Markov model-based method that estimates the probability that each nucleotide belongs to a conserved element, based on the multiple

alignments. It considers not just each individual alignment column, but also its flanking columns. PhastCons is sensitive to runs of conserved sites, and therefore effective for identifying conserved elements [17-20]. The predicted conserved elements are assigned a log-odds score, ranging from 0 to 1000, equals to their log probability under the conserved model minus their log probability under the non-conserved model. According to UCSC Genome Browser, the default phastCons parameters used were: expected-length=45, target-coverage=0.3, rho=0.3 (https://genome.ucsc.edu/cgibin/hgTrackUi?hgsid=493737591_a0L8Go632aHEn5NYvS5rUss7smqh&c=chr10&g=cons100way).

We looked for CRMs in the conserved sequences as identified by the phastCons algorithm. In particular, to implement the conserved elements to CRM prediction, we searched the co-occurrence of conserved elements and putative CRMs with the average means of negative selection probability above 0.2 (from 0 to 1 scale) from 100% coverage of CRM DNA sequences.

Next, putative *cis*-regulatory modules (CRMs) were searched, coinciding with the most conserved sequence elements. In general (for a review see Hardison *et al.* [21]), three major approaches are used (alone or in combination) to predict CRMS: the first is to search genomic DNA for clusters of transcription-factor binding-site motifs, A second approach for identifying CRMs involves the identification of noncoding DNA sequences under evolutionary constraint and a third approach directly assays for DNA sequences having epigenetic features characteristic of regulatory regions.

The MatchTM program or FIMO application5 were used to scan the conserved sequence elements for *TFBS* associated with high tissue- specific expression[5]. In a final filtering step, candidate *Sk-CRMs* were mapped against the human hg19 genome using the blat tool at the UCSC genome browser (https://genome.ucsc.edu). Next, the mapped regions were visualized along the ENCODE Regulation supertrack containing information relevant to regulation of transcription from the ENCODE project [22]. The layered H3K4Me1 and layered H3K27Ac

marks are epigenetic signatures often found near regulatory elements, while the DNase I Hypersensitivity tracks indicate where chromatin is hypersensitive to cutting by the DNase enzyme and are associated with both regulatory regions and promoters. With respect to their genomic locations they appear highly correlated and often coincide at active regulatory elements but the overlaps are far from perfect and therefore we additionally filter for regions where the overlaps are maximal with the candidate CRMs. The epigenetic marks of histone modification used in CRM design are available in UCSC genome browser, which were taken from only human skeletal muscle myoblast (HSMM) from ENCODE project. These assays are relatively independent of each other, so overlap between candidate *Sk-CRMs* and these quintessential epigenetic signatures increase the likelihood that a given *Sk-CRM* enhances transcription. By using internally developed Perl scripts, we identified highly conserved *CRMs* containing clusters of *TFBS* putatively associated with high tissue-specific expression.

AAV vector production and purification

AAV serotype 9 (AAV9) is well suited to achieve efficient transduction in heart and skeletal muscle [23-25]. Consequently, all AAV vectors used in this study were produced using this serotype. Briefly, calcium phosphate (Invitrogen Corp, Carlsbad, CA, USA) co-transfection of AAV-293 cells (#240073; Stratagene/Agilent, USA) with the AAV plasmid of interest, a chimeric packaging construct and an adenoviral helper plasmid, was used to produce AAV vectors [25]. Cells were harvested 2 days after transfection and lysed by freeze/thaw cycles and sonication, followed by benzonase (Novagen, Madison, WI, USA) and deoxycholic acid (Sigma-Aldrich, St Louis, MO, USA) treatments and 3 consecutive rounds of cesium chloride (Invitrogen Corp, Carlsbad, CA, USA) density gradient ultracentrifugation. Fractions containing the AAV vector particles were collected and dialyzed in Dulbecco's phosphate buffered saline (PBS) (Gibco, BRL) containing 1 mM $MgCl_2$. Quantitative (q) real-time PCR with SYBR® Green and primers specifically designed for the *Luc* and *MD1* genes were used to determine vector titers. The forward and reverse *Luc*-specific primers used were 5'-CCCACCGTCGTATTCGTGAG-3' and 5'-TCAGGGCGATGGTTTTGTCCC-3', respectively. The forward and reverse *MD1*-specific primers were 5'-GTGCCCTACTACATCAA-3' and 5'-

AGGTTGTGCTGGTCCA-3', respectively. To generate standard curves, known copy numbers of the corresponding vector plasmids were used. All vectors attained normal titers exceeding 10^{11} vg/ml, except for the vector containing *Sk-CRM5,* which was therefore not retained for the subsequent comprehensive *in vivo* characterizations.

Transduction efficiency and vector biodistribution

Transduction efficiency and biodistribution was evaluated by quantifying *Luc* transgene copy numbers in the different organs and tissues [26]. Briefly, genomic DNA was extracted from 30 mg of each tissue according to DNeasy Blood & Tissue Kit protocol (Qiagen, Chatsworth, CA, USA) and 100 ng of genomic DNA from each sample was subjected to qPCR, using the *Luc*-specific forward primer 5'-CCCACCGTCGTATTCGTGAG-3' and reverse primer 5'-TCAGGGCGATGGTTTTGTCCC-3' (amplicon 217 bp). The qPCR results were expressed as mean AAV copy number/100 ng of genomic DNA. Known copy numbers (10^2 - 10^7) of the corresponding plasmid were serially diluted and used to generate the standard curve.

mRNA analysis

Total RNA was extracted from different tissues of mice injected with the various AAV vectors using a silica-membrane based purification kit according to the manufacturer's instructions (Invitrogen Corp, Carlsbad, CA, USA). Subsequently, 100 ng of total RNA from each sample was subjected to reverse transcription (RT) using a cDNA synthesis kit (Invitrogen Corp, Carlsbad, CA, USA). Next, a cDNA amount corresponding to 100 ng of total RNA was amplified by quantitative qPCR on an ABI 7700 (Applied Biosystems, Foster City, CA, USA). To quantify *Luc* mRNA levels, *Luc*-specific forward (5'-CCCACCGTCGTATTCGTGAG-3') and reverse (5'-TCAGGGCGATGGTTTTGTCCC-3') primers were used, generating a 217 bp amplicon. For the phenotypic correction studies (see below), *MD1* mRNA levels were quantified using the same approach using forward 5'-GTGCCCTACTACATCAA-3' and reverse 5'-AGGTTGTGCTGGTCCA-3' primers, generating a 206 bp amplicon. Similarly, to determine *FST-2A-Luc* mRNA levels, forward 5'-CCCACCGTCGTATTCGTGAG-3' and reverse 5'-TCAGGGCGATGGTTTTGTCCC-3' primers were used, yielding a 217 bp amplicon.

The *Luc*, *MD1* and *FST* mRNA levels were normalized to mRNA levels of the endogenous murine glyceraldehyde-3-phosphate dehydrogenase (*mGapdh*) gene, using 5'-TGTGTCCGTCGTGGATCTGA-3' and 5'-GCCTGCTTCACCACCTTCTTGA-3' as forward and reverse primers, respectively (amplicon 82 bp). RNA samples were amplified with and without reverse transcriptase to exclude DNA amplification. ΔC_t was calculated by subtracting the C_t of the control gene from the C_t of the gene of interest for each tissue (heart, gastrocnemius and quadriceps). The ΔC_t of the control tissue sample was subtracted from the ΔC_t of the corresponding experimental tissue sample and the results were graphically represented as $\Delta\Delta C_t$ for each tissue of different treated groups (MD1, FST and MD1+FST).

Chromatin immunoprecipitation assay (ChIP assay)

Neonatal mice injected intravenously with ssAAV9-Sk-CRM4-Des-Luc (5×10^9 vg/per mouse) were euthanized 4 weeks post vector-injection. Heart and gastrocnemius muscle tissues were harvested and submersed in PBS with 1% formaldehyde, cut into small pieces and incubated at room temperature for 15 minutes. Fixation was stopped by the addition of 0.125 M glycine. The tissue pieces were then homogenized with a Tissue Tearer (BioSpec Products, Virginia Ave, Bartlesville, USA) and spun down and washed twice in PBS. Chromatin was isolated by the addition of lysis buffer, followed by disruption with a Dounce homogenizer. Lysates were sonicated and the DNA sheared to an average length of 300-500 bp. Genomic DNA was prepared by treating aliquots of chromatin with RNase, proteinase K and heat for de-crosslinking, followed by ethanol precipitation. Pellets were resuspended and the resulting DNA was quantified on a NanoDrop spectrophotometer. Extrapolation to the original chromatin volume allowed quantitation of the total chromatin yield. An aliquot of chromatin (30 μg) was pre-cleared with protein agarose beads (Invitrogen, Waltham, MA, USA). Genomic DNA regions of interest were isolated using 4 μg of SRF-specific antibody (sc-335; Santa Cruz Biotechnology, Santa Cruz, CA, USA) or a CEBP-specific antibody (sc-150; Santa Cruz Biotechnology, Santa Cruz, CA, USA). Complexes were washed, eluted from the beads with SDS buffer, and subjected to RNase and proteinase K treatment. Crosslinks were reversed by incubation overnight at 65 °C, and ChIP DNA was purified by phenol-chloroform extraction

and ethanol precipitation. Quantitative PCR (qPCR) reactions were carried out in triplicate on specific genomic regions using SYBR Green Supermix (Bio-Rad). The resulting signals were normalized for primer efficiency by carrying out quantitative PCR for each primer pair using the genomic input DNA with *Sk-CRM4*-specific forward (5'-GTCCCTCACTCCCAACTCAG-3') and reverse (5'- GAGGAGAAGGAGATCAGACACTG-3') primers. Negative control primers were purchased from Active Motif (#71012; Carlsbad, CA, USA) and are specific for non-transcribed gene sequences on chromosome 17.

Histological analysis

Mice injected with the ssAAV9-Sk-CRM4-Des-MD1 and ssAAV9-Sk-CRM4-Des-FST vectors (2×10^{10} vg per mouse) were euthanized 18 weeks post vector injection and the gastrocnemius muscles were harvested and fixed overnight in 4% paraformaldehyde at 4°C and then stored in 70% ethanol. The fixed tissues were then embedded in paraffin. Five μm transverse sections were prepared for hematoxylin and eosin staining. The tissue sections were first dewaxed and rehydrated by treatment with toluene, twice, for 5 min each time, followed by absolute isopropanol, then 90% isopropanol and finally 70% isopropanol, each for 1 minute. The tissue sections were then stained with hematoxylin (Sigma Aldrich, St. Louis, Missouri, USA) for 10 min, washed under running water for 5 min and then stained with 1% erythrosine (Sigma Aldrich, St. Louis, Missouri, USA) for 5 min followed by a brief wash under running water. Dehydration of the tissue sections was done by treatment with 70% isopropanol, 90% isopropanol and absolute isopropanol followed by toluene for a few seconds. Microscopic analysis was done upon mounting the sections using Pertex (Histolab, Sweden) as the mounting medium. Nine random fields under 20x magnification from the largest tissue sections were chosen and the number of fibers with central nuclei was counted. The percentage of central nucleation in muscle fibers per condition was calculated by using the formula: 100 x (number of centrally nucleated fibers/total number of fibers per field). The fiber cross-sectional area of each muscle fiber for the different groups, both treated (MD1, FST, MD1+FST) and untreated (SCID/mdx and C57/BL6) was measured by using the ImageJ software. Eight fields were considered for each condition and the fiber cross sectional area of

the total number of fibers in each of these fields for the different groups were represented graphically.

For immunofluorescence staining, the heart and the TA muscles were embedded in Optimal Cutting Temperature compound (OCT; Thermo Scientific, Waltham, MA, USA) and snap frozen in liquid nitrogen cooled isopentane and 6 μm thick sections were cut using a MicromHM550 cryostat (Thermo Scientific, Waltham, MA, USA). Frozen tissue sections were thawed in PBS (with Ca^{2+}, Mg^{2+}) for 10 min followed by a brief wash with PBS for 5 min. The tissue sections were then washed in PBS with 0.2% triton and 1% BSA and incubated with blocking serum containing 20% donkey serum in PBS with 0.2% triton and 1% BSA. The tissue sections were then incubated overnight at 4°C with the following primary antibodies: rabbit anti-mouse laminin from (ab11575; Abcam, Cambridge, UK) and mouse monoclonal anti-human dystrophin (NCL-DYS3; Novocatsra, Newcastle, UK). The samples were subsequently washed three times with PBS and then incubated with the appropriate fluorescein-isothyocyanate (FITC) or tetramethylrhodamine-isothiocyanate-conjugated (TRITC) anti-mouse or anti-rabbit (1:500, Life Technologies, Carlsbad, CA, USA) and 4',6-diamidino-2-phenylindole (DAPI; Hoechst nucleic acid stain, Life technologies, USA) (1:1000 dilution) for 1 hour at room temperature. After three final washes, the coverslips were mounted on glass slides using the Fluorosave mounting medium (Dako, Denmark) and analyzed under a fluorescent microscope (Nikon Eclipse 80i) at 20x magnification.

Supplementary References

1 Wittkopp, P. J. & Kalay, G. Cis-regulatory elements: molecular mechanisms and evolutionary processes underlying divergence. *Nat. Rev. Genet.* **13**, 59-69 (2011).

2 Hardison, R. C. & Taylor, J. Genomic approaches towards finding cis-regulatory modules in animals. *Nat. Rev. Genet.* **13**, 469-483 (2012).

3 Spitz, F. & Furlong, E. E. Transcription factors: from enhancer binding to developmental control. *Nat. Rev. Genet.* **13**, 613-626 (2012).

4 Chuah, M. K. *et al.* Liver-Specific Transcriptional Modules Identified by Genome-Wide In Silico Analysis Enable Efficient Gene Therapy in Mice and Non-Human Primates. *Mol. Ther.* **22**, 1605-1613 (2014).

5 Johansson, O., Alkema, W., Wasserman, W. W. & Lagergren, J. Identification of functional clusters of transcription factor binding motifs in genome sequences: the MSCAN algorithm. *Bioinformatics* **19 Suppl 1**, i169-176 (2003).

6 Busser, B. W., Haimovich, J., Huang, D., Ovcharenko, I. & Michelson, A. M. Enhancer modeling uncovers transcriptional signatures of individual cardiac cell states in Drosophila. *Nucleic Acids Res.* **43**, 1726-1739 (2015).

7 Girgis, H. Z. & Ovcharenko, I. Predicting tissue specific cis-regulatory modules in the human genome using pairs of co-occurring motifs. *BMC Bioinformatics* **13**, 25 (2012).

8 Narlikar, L. *et al.* Genome-wide discovery of human heart enhancers. *Genome Res.* **20**, 381-392 (2010).

9 Liu, R., Hannenhalli, S. & Bucan, M. Motifs and cis-regulatory modules mediating the expression of genes co-expressed in presynaptic neurons. *Genome Biol.* **10**, R72 (2009).

10 Liu, X., Yu, X., Zack, D. J., Zhu, H. & Qian, J. TiGER: a database for tissue-specific gene expression and regulation. *BMC Bioinformatics* **9**, 271 (2008).

11 Marino-Ramirez, L., Spouge, J. L., Kanga, G. C. & Landsman, D. Statistical analysis of over-represented words in human promoter sequences. *Nucleic Acids Res* **32**, 949-958 (2004).

12 Trinklein, N. D., Aldred, S. J., Saldanha, A. J. & Myers, R. M. Identification and functional analysis of human transcriptional promoters. *Genome Res* **13**, 308-312 (2003).

13 Grant, C. E., Bailey, T. L. & Noble, W. S. FIMO: scanning for occurrences of a given motif. *Bioinformatics* **27**, 1017-1018 (2011).

14 De Bleser, P., Hooghe, B., Vlieghe, D. & van Roy, F. A distance difference matrix approach to identifying transcription factors that regulate differential gene expression. *Genome Biol* **8**, R83 (2007).

15 Blanchette, M. *et al.* Aligning multiple genomic sequences with the threaded blockset aligner. *Genome Res* **14**, 708-715 (2004).

16 Siepel, A. *et al.* Evolutionarily conserved elements in vertebrate, insect, worm, and yeast genomes. *Genome Res* **15**, 1034-1050 (2005).

17 Felsenstein, J. & Churchill, G. A. A Hidden Markov Model approach to variation among sites in rate of evolution. *Mol Biol Evol* **13**, 93-104 (1996).

18 Margulies, E. H., Blanchette, M., Program, N. C. S., Haussler, D. & Green, E. D. Identification and characterization of multi-species conserved sequences. *Genome Res* **13**, 2507-2518 (2003).

19 Mayor, C. *et al.* VISTA : visualizing global DNA sequence alignments of arbitrary length. *Bioinformatics* **16**, 1046-1047 (2000).

20 Yang, Z. A space-time process model for the evolution of DNA sequences. *Genetics* **139**, 993-1005 (1995).

21 Hardison, R. C. & Taylor, J. Genomic approaches towards finding cis-regulatory modules in animals. *Nat Rev Genet* **13**, 469-483 (2012).

22 Consortium, E. P. An integrated encyclopedia of DNA elements in the human genome. *Nature* **489**, 57-74 (2012).

23 Inagaki, K. *et al.* Robust systemic transduction with AAV9 vectors in mice: efficient global cardiac gene transfer superior to that of AAV8. *Mol Ther* **14**, 45-53 (2006).

24 Pacak, C. A. *et al.* Recombinant adeno-associated virus serotype 9 leads to preferential cardiac transduction in vivo. *Circ Res* **99**, e3-9 (2006).
25 Vandendriessche, T. *et al.* Efficacy and safety of adeno-associated viral vectors based on serotype 8 and 9 vs. lentiviral vectors for hemophilia B gene therapy. *J Thromb Haemost* **5**, 16-24 (2007).
26 Valouev, A. *et al.* Genome-wide analysis of transcription factor binding sites based on ChIP-Seq data. *Nat Methods* **5**, 829-834 (2008).
