# Supplementary material for: Next-generation muscle-directed gene therapy by in silico vector design
Source: Nat Commun. 2019 Jan 30;10:492. doi: 10.1038/s41467-018-08283-7 (PMC6353880; doi:10.1038/s41467-018-08283-7)
Supplement: Supplementary file 2 — Reporting Summary [file 41467_2018_8283_MOESM2_ESM.pdf]

nature research

Corresponding author(s): Dr. Thierry VandenDriessche & Dr. Marinee K.L. Chuah

# Reporting Summary

Nature Research wishes to improve the reproducibility of the work that we publish. This form provides structure for consistency and transparency in reporting. For further information on Nature Research policies, see Authors & Referees and the Editorial Policy Checklist.

## Statistical parameters

When statistical analyses are reported, confirm that the following items are present in the relevant location (e.g. figure legend, table legend, main text, or Methods section).

| n/a | Confirmed | |
|---|---|---|
| ☐ | ☒ | The exact sample size (*n*) for each experimental group/condition, given as a discrete number and unit of measurement |
| ☐ | ☒ | An indication of whether measurements were taken from distinct samples or whether the same sample was measured repeatedly |
| ☐ | ☒ | The statistical test(s) used AND whether they are one- or two-sided<br>*Only common tests should be described solely by name; describe more complex techniques in the Methods section.* |
| ☒ | ☐ | A description of all covariates tested |
| ☒ | ☐ | A description of any assumptions or corrections, such as tests of normality and adjustment for multiple comparisons |
| ☐ | ☒ | A full description of the statistics including central tendency (e.g. means) or other basic estimates (e.g. regression coefficient) AND variation (e.g. standard deviation) or associated estimates of uncertainty (e.g. confidence intervals) |
| ☐ | ☒ | For null hypothesis testing, the test statistic (e.g. *F*, *t*, *r*) with confidence intervals, effect sizes, degrees of freedom and *P* value noted<br>*Give P values as exact values whenever suitable.* |
| ☒ | ☐ | For Bayesian analysis, information on the choice of priors and Markov chain Monte Carlo settings |
| ☒ | ☐ | For hierarchical and complex designs, identification of the appropriate level for tests and full reporting of outcomes |
| ☒ | ☐ | Estimates of effect sizes (e.g. Cohen's *d*, Pearson's *r*), indicating how they were calculated |
| ☐ | ☒ | Clearly defined error bars<br>*State explicitly what error bars represent (e.g. SD, SE, CI)* |

*Our web collection on statistics for biologists may be useful.*

## Software and code

Policy information about availability of computer code

| | |
|---|---|
| Data collection | M3 Vision software (BiospaceLab), NIS-Elements AR software (Nikon), ImageQuant LAS4000 (GE Healthcare). |
| Data analysis | GraphPad Prism 6 (GraphPad Software), ImageJ (National Institutes of Health), Excel (Microsoft) |

For manuscripts utilizing custom algorithms or software that are central to the research but not yet described in published literature, software must be made available to editors/reviewers upon request. We strongly encourage code deposition in a community repository (e.g. GitHub). See the Nature Research guidelines for submitting code & software for further information.

## Data

Policy information about availability of data

All manuscripts must include a data availability statement. This statement should provide the following information, where applicable:

- Accession codes, unique identifiers, or web links for publicly available datasets
- A list of figures that have associated raw data
- A description of any restrictions on data availability

The authors declare that all data supporting the findings of this investigation are available within the article, its Supplementary Information, and from the corresponding authors, upon reasonable request. All comprehensive Western blot images including the molecular weight markers were presented in Supplementary Figure 6.

# Field-specific reporting

Please select the best fit for your research. If you are not sure, read the appropriate sections before making your selection.

☒ Life sciences ☐ Behavioural & social sciences ☐ Ecological, evolutionary & environmental sciences

For a reference copy of the document with all sections, see nature.com/authors/policies/ReportingSummary-flat.pdf

# Life sciences study design

All studies must disclose on these points even when the disclosure is negative.

| | |
|---|---|
| Sample size | Sample size was chosen depending on the technique used and based on our experience (Methods/Quantification and Statistical Analysis). No power calculations were performed to choose group size (Methods/Mice). |
| Data exclusions | All samples were included in analysis (Methods/mice). No data were excluded. |
| Replication | Every experiment was repeated and reproduced at least two times, and where appropriate the representative result is presented. The number of independent experiments and also the number of biological replicates for each chart is stated in the statistical analysis section. |
| Randomization | For each cohorts of various experiments, mice were randomly selected from a group of mice of same age and similar weight to standardize control and experimental groups as much as possible. |
| Blinding | Mice and samples were coded based on the ear tag number. The ear tag number was decoded after conducting the experiment. The designated investigator injecting the vectors was blinded to the experimental outcome. The investigators were blinded to the precise nature of the CRMs or vectors. The screening of the CRMs and testing of the vectors was therefore blinded. |

# Reporting for specific materials, systems and methods

## Materials & experimental systems

| n/a | Involved in the study |
|---|---|
| ☒ | ☐ Unique biological materials |
| ☐ | ☒ Antibodies |
| ☐ | ☒ Eukaryotic cell lines |
| ☒ | ☐ Palaeontology |
| ☐ | ☒ Animals and other organisms |
| ☒ | ☐ Human research participants |

## Methods

| n/a | Involved in the study |
|---|---|
| ☒ | ☐ ChIP-seq |
| ☒ | ☐ Flow cytometry |
| ☒ | ☐ MRI-based neuroimaging |

## Antibodies

| | |
|---|---|
| Antibodies used | Anti-SRF (sc-335, Santa Cruz Biotechnology), Anti-CEBP (sc-150, Santa Cruz Biotechnology) Anti dystrophin (NCL-DYS3, Novocatsra), Anti-GAPDH (2118; Cell Signaling ), Anti-CD4 (Cell signaling) Anti-CD8 (Cell signaling), Anti-Laminin (ab11575, Abcam), FITC-conjugated anti-mouse IgG (Life Technologies), TRITC -conjugated anti-rabbit IgG (Life Technologies), HRP-conjugated anti-rabbit IgG (Cell Signaling), HRP-conjugated anti-mouse IgG (Cell Signaling). |
| Validation | All antibodies used in the study are commercially available and have been validated by the manufacturer. |

## Eukaryotic cell lines

Policy information about cell lines

| | |
|---|---|
| Cell line source(s) | AAV-293 cells (HEK-293) (not listed in ICLAC register; see below) |
| Authentication | Cell line is commercially available from Stratagene/Agilent http://www.integratedsci.com.au/product/aav-293-cells.html Catalog Code: 240073 |
| Mycoplasma contamination | Cell line was not tested for mycoplasma contamination |

| Commonly misidentified lines (See ICLAC register) | N/A |
|---|---|

## Animals and other organisms

Policy information about studies involving animals; ARRIVE guidelines recommended for reporting animal research

| | |
|---|---|
| Laboratory animals | C57BL/6, CB17/IcrTac/Prkdcscid, mdx, SCID/mdx mice were housed in the animal facility in an IVC system. Littermates of the same sex were randomly assigned to experimental groups (3 or 5 mice per group) The mice with normal health and immune status were used for the experiments. |
| Wild animals | The study did not involve wild animals. |
| Field-collected samples | The study did not involve field-collected samples. |
